# c-Jun N-Terminal Kinase: A Spatiotemporal Regulator of Cell Fate and Function

**DOI:** 10.3390/biology15131009

**Published:** 2026-06-25

**Authors:** Seth Thesing, Mohammed Salahuddin, Emily Okonek, Ryan L. Hanson

**Affiliations:** Department of Biology & Microbiology, College of Natural Sciences, South Dakota State University, Brookings, SD 57007, USA; seth.thesing@sdstate.edu (S.T.); mohammed.salahuddin@sdstate.edu (M.S.); emily.okonek@sdstate.edu (E.O.)

**Keywords:** JNK, cell fate, gene expression, spatial localization, temporal dynamics

## Abstract

c-Jun N-terminal kinase (JNK) is a highly conserved, stress-activated protein kinase that plays key roles in cellular development and cell fate. An extensive study over more than 30 years has identified roughly 100 substrates for this kinase including the transcription factor c-Jun and other cell fate effectors. These studies have shown that JNK activation is tightly regulated both spatially through recruitment to subcellular locations and temporally through specific activation dynamics. Ultimately, these two regulatory mechanisms contribute to JNK’s function as a major driver of cell fate and function. A growing field of live-cell imaging, biosensor development, and other novel approaches to manipulate kinase function and localization are now providing novel insights into JNK function at the single-cell level. The purpose of this review is to illustrate our historical understanding of the spatiotemporal functions of JNK signaling within cells as well as emerging studies within the field. Ultimately, we aim to provide insight into remaining knowledge gaps within the field and how emerging technologies may help address these questions.

## Simple Summary

c-Jun N-terminal kinase (JNK) is a critical regulator of cellular stress responses within our cells. Extensive biochemical and in vivo characterization in the 1990s and 2000s provided significant insight into the substrates, regulatory networks, physiological relevance, and function of JNK in the determination of cell fate. These studies provide the foundation for ongoing research in the field today. Over the past decade, advances in microscopy and biosensor development have provided novel insights into the function of JNK both through time and space within cells. Critically, these studies have illustrated how the single-cell dynamics of JNK activation contribute to differential cell fates within cell populations and the mechanisms driving these patterns. The goal of this review is to highlight our current understanding of JNK as a spatiotemporal regulator of cell function and identify remaining knowledge gaps and emerging research areas that will provide further insight into the cellular functions of this critical cellular kinase.

## 1. Introduction

Initially identified as a microtubule-associated kinase [1], c-Jun N-terminal kinase (JNK) or stress-activated protein kinase (SAPK) has been extensively studied over multiple decades. Stress-activated protein kinases are highly conserved with homologs spanning yeast, *Drosophila*, and ultimately multiple isoforms within mammalian systems (JNK1/2/3) [2]. Early seminal studies of JNK include the identification of the major substrate c-Jun, demonstration of the in vivo relevance of JNK, and identification of the array of stimuli that drive JNK activation [3]. Among the most well-studied functions of JNK is its ability to mediate the balance of both cell death and cell survival as a stress-activated protein kinase. A key underlying feature of this regulation is the notion of temporal regulation (i.e., how a pathway is activated over time). Early studies demonstrated that a transient or short pulse of JNK activation was associated with cell survival, whereas a transition to a prolonged, sustained activation was associated with the induction of cell death [4,5]. With the growing advent of live-cell imaging methods and biosensor development, numerous groups have demonstrated that temporal dynamics play critical roles across the spectrum of stress-responsive pathways; including p53, NF-kB, and FOXO [6,7,8,9]. These same approaches have provided tremendous insight into the regulation and function of JNK at the single cell level, which we highlight in this review.

Beyond temporal regulation, research has also shown that the spatial localization (i.e., where JNK is within the cells) adds an additional regulatory layer to the function of JNK. Notably, the recruitment of JNK to the outer mitochondrial membrane (OMM) via the scaffold protein Sab appears to be required for the prolonged activation of JNK [10]. However, JNK has been shown to localize to numerous cellular compartments, including the nucleus, cytoskeleton, and stress granules. The purpose of this review is to summarize our current understanding of the molecular networks that mediate JNK signaling, how these impact both the temporal and spatial function of JNK, and ultimately how these dynamics contribute to JNK’s biological function. Within this paper, we highlight remaining gaps within the study of spatiotemporal JNK biology and posit how emerging technologies and methodologies may promote further interrogation of this critical signaling network.

## 2. Network Architecture and Regulation of JNK Signaling

JNK is a member of the mitogen-activated protein kinase (MAPK) family [11]. As in other MAPK signaling networks, JNK is primarily regulated as part of a three-kinase cascade (MAP3K, MAP2K, MAPK) which ultimately results in activating the dual phosphorylation of threonine and tyrosine residues of JNK within the highly conserved TPY motif [11]. The phosphorylation and dephosphorylation of this motif, typically driven by dual-specificity MAPK phosphatases (MKPs), is one of the primary mechanisms of JNK regulation [12]. However, other regulatory mechanisms include the spatial sequestration and degradation of JNK or its downstream substrates. Within this section, we provide a brief overview of the classical MAPK cascade which regulates JNK as well as known negative regulators that shape the activation kinetics of JNK in response to cellular stimuli before concluding with a summary of known downstream substrates of JNK signaling that function as the effectors of JNK-mediated cellular responses. It should be noted that within mammalian cells, JNK consists of three distinct genes, JNK1 [13], JNK2 [14], and JNK3 [15]. JNK1 and 2 are ubiquitously expressed in most tissues; however, JNK3 expression is restricted to the heart, brain, and testes [11]. While each of these isoforms exhibit significant homology and redundancy in some functions, studies have shown differences in activity among JNK1, 2, and 3. For example, JNK2 exhibits higher affinity for c-Jun [14], JNK1 and JNK2 exhibit differential effects on fibroblast proliferation [16], and JNK3 is a driver of neuronal cell death [17,18]. A summary of some known differences between these genes is highlighted in Table 1. However, the activation of these three variants occurs through similar mechanisms and regulators as discussed in the following sections.

### 2.1. Regulation of JNK Through the MAPK Cascade

**Figure 1:**
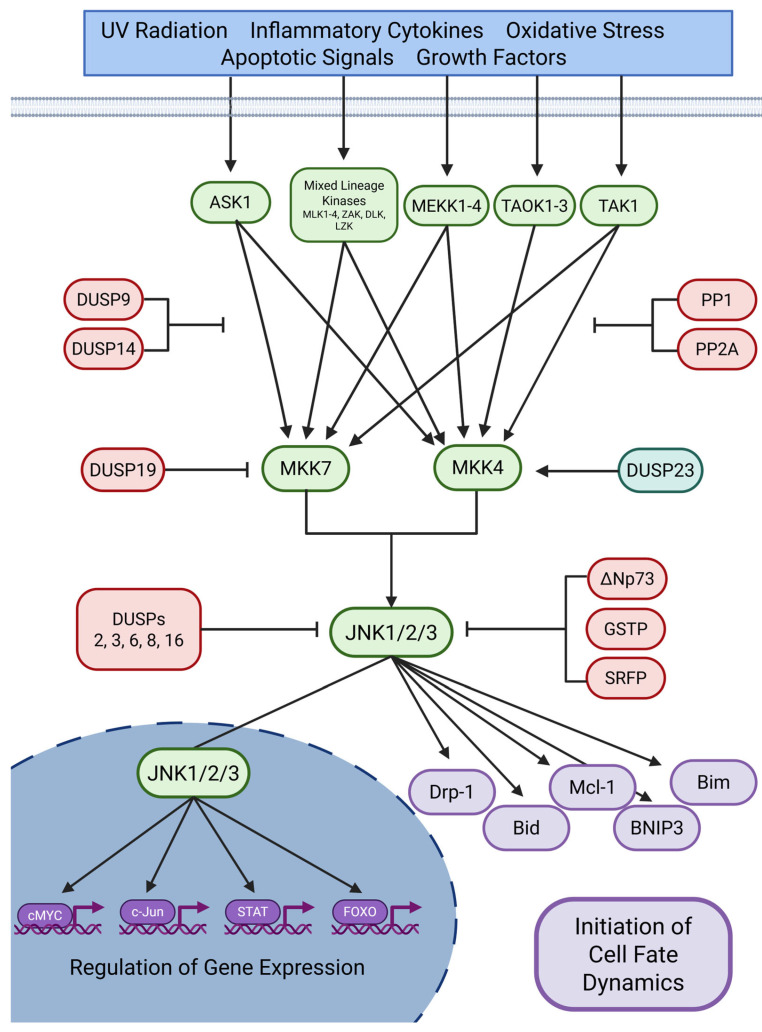
JNK signaling cascade. Schematic overview of the general JNK signaling cascade. Upstream MAP3Ks are activated in response to an array of stimuli, including UV-light and oxidative stress, and relay signals downstream through MKK4 and MKK7 to JNK. JNK can then phosphorylate both nuclear and non-nuclear substrates to shape gene expression and cell fate. Inactivation is driven by an array of phosphatases (DUSPs and PPs) as well as additional regulators.

Members of the MAPK family are primarily regulated through a three-kinase signaling module (MAP3K, MAP2K, and MAPK) that is highly conserved across a range of species from yeast to humans (Figure 1) [26]. Typically, these cascades are oriented in association with specific scaffold proteins to facilitate activation [27]. In mammalian cells, the MAPK pathways consist of the ERK, p38, and JNK signaling networks; however, the activation mechanisms across these three pathways are largely similar. Upon initial stimulation, the initiating kinase of this cascade, MAP3K (MAP kinase kinase kinase), becomes active through a variety of mechanisms including recruitment to specific receptors [28], relief of autoinhibition [29,30], dimerization [31], and phosphorylation [11,32]. While the repertoire of MAP2K and JNK are relatively limited, significant diversity exists in the families of MAP3K that can ultimately regulate the activation of JNK. These range from apoptosis-stimulating kinases (ASK1-3), mixed lineage kinases (MLK1-4, DLK, LZK, and ZAK), MEKK1-4, TAK1, and TAOK1-3 [33]. This range of diversity potentially allows the specific activation of JNK and other pathways in response to a diverse range of stimuli [34]. An ongoing question within the field is how these diverse MAP3Ks function in driving specific downstream responses, and emerging studies using MAP3K profiling are beginning to highlight key roles for these kinases [35]. For example, ZAK is a critical sensor of ribotoxicity. In unstressed conditions, ZAK remains associated with ribosomes, but upon ribosome disruption, via collisions or stalling, ZAK undergoes autophosphorylation and is released to initiate the downstream activation of JNK [36,37]. In contrast, TNFα, binding to the TNF receptor, can recruit ASK1 via TRAF2 to promote dimerization and activation by relieving its repression by 14-3-3 and Trx-1 binding [28,38,39]. Once active, ASK1 can relay the signal to the MAP2Ks, which can then phosphorylate and activate JNK. Recent studies also implicate MAP3K5 (ASK1) as a regulator of pyroptosis via JNK [40].

Once MAP3Ks have become active, they can then phosphorylate and activate the downstream MAP2Ks (MAP kinase kinase). Specifically, JNK is activated via two MAP2Ks, MKK4 and MKK7. MKK7 is considered a specific activator of JNK [41], while MKK4 coordinates the activation of both JNK and p38 [42]. Similar to MAP3Ks providing specificity in activation, MKK4 and MKK7 appear to regulate distinct responses. For example, MKK7 is primarily activated in response to cytokine signaling and regulates JNK-mediated cytokine production [43,44]. Once activated, MKK4 and MKK7 will activate JNK via dual phosphorylation of the threonine and tyrosine residues within the TPY motif. Interestingly, while both MKK7 and MKK4 can phosphorylate JNK, they appear to work synergistically with one another, as MKK7 shows stronger preferences for phosphorylation of the threonine site, while MKK4 favors the tyrosine site [41]. While MKK4 and MKK7 are the classical activators of JNK, emerging evidence suggests that the MAPK cascade may be more flexible than initially thought, as the MAP2K MKK3, originally thought to be specific to p38 activation, has been shown to induce JNK activity in *Drosophila* [45]. Therefore, despite extensive study, a further interrogation of network architecture and signaling crosstalk is needed to effectively parse out the regulation of JNK via the MAPK cascade.

### 2.2. Negative Regulation of JNK Signaling

While the phosphorylation of JNK within the TPY motif represents the mechanism of JNK activation, dephosphorylation of this same motif acts as the primary mechanism of negative regulation. This dephosphorylation is catalyzed by dual-specificity phosphatases (DUSPs), which are also termed mitogen-activated protein kinase phosphatases (MKPs). These phosphatases can directly regulate JNK by dephosphorylation of the critical threonine and tyrosine sites or indirectly by acting as scaffolds. While the DUSP family of proteins contains several members, many of these proteins are localized to specific cellular compartments and exhibit preferences for specific MAPKs [46]. The direct dephosphorylation of JNK occurs via DUSP1, 2 [47,48], 3 [49], 6 [50], 8 [51], and 16 [52]. JNK activation is also subject to DUSP regulation through indirect mechanisms. DUSP9 and DUSP14 inhibit the activation of upstream MAP3Ks, which prevents signal propagation to the MAP2Ks. DUSP9 inactivates ASK1 [53], and DUSP14 interferes with TAK1 activation through dephosphorylation of the TAK1 binding protein TAB1 [54]. Scaffolding DUSPs also regulate JNK activation through upstream targets; however, these effects can be more complex than simple inactivation. For example, DUSP19 interacts with MKK7, and early studies found inhibitory effects on downstream JNK activation [55]; however, this effect may be concentration dependent, as at higher doses, DUSP19 potentiates signaling through JNK2 [56]. DUSP23 is another scaffolding protein that tethers MKK4 and JNK. However, instead of inhibiting JNK activation, it acts as a positive regulator [57].

Outside of DUSPs, JNK has been shown to be regulated via a number of other interaction partners, including Glutathione S-Transferase Pi (GTSP) [58], an isoform of p73, ΔNp73 [59], and SRFP [60]. However, precise molecular mechanisms behind some of these regulators are unclear. Beyond the direct regulation of JNK, the downstream regulation of effector molecules can further shape JNK-mediated responses.

### 2.3. Effectors of JNK Function: Downstream Substrates

Once activated, JNK phosphorylates serine and threonine residues on target proteins in a proline-directed manner with a consensus sequence of PX(S/T)P [61]. These target proteins span an array of cellular compartments, including the cytoskeleton [62], mitochondria [63,64,65], and the nucleus where JNK phosphorylates numerous transcription factors, including c-Jun on serine 63 and serine 73, to drive gene expression [66,67,68]. Currently, the number of known JNK substrates is estimated to be around 100 unique proteins [69]. As a result of the diverse localization of these substrates, JNK can drive a variety of downstream cellular responses. This includes the regulation of cell death and apoptosis through the direct phosphorylation of pro-apoptotic proteins like Bim [65] and Bid [70], the indirect release of pro-apoptotic factors like Bax by the phosphorylation of repressors [71], and the phosphorylation of anti-apoptotic proteins like Mcl-1 [72]. JNK also regulates mitochondrial fission through the phosphorylation of Drp-1 [63] and mitophagy through the phosphorylation of BNIP3 [64]. Finally, the regulation of gene expression by JNK is another major output of downstream effectors. This is commonly thought to be driven by c-Jun/AP-1, but other transcription factors like p53 [67], c-Myc [68], STAT [73], and FOXO [74] are regulated via JNK. With this wide array of potential downstream responses driven by JNK activation, cells must have a mechanism for providing specificity to these responses driven by JNK. Within the remaining sections of this review, we focus on two regulatory mechanisms. The first is the temporal dynamics of how JNK is activated over time, while the second is the spatial localization that limits where JNK is active.

## 3. Temporal Dynamics of JNK in the Regulation of Cell Fate and Function

While the core biochemical architecture of the JNK signaling network is well established, existing and emerging evidence demonstrates that the temporal dynamics of JNK activation, such as the duration of activity over time, are critical determinants of downstream cellular responses, including both cell fate and gene expression. Within this section, we highlight both the foundational work that demonstrated roles for JNK dynamics in cell fate determination as well as emerging findings leveraging advances in microscopic imaging that further support and expand upon this work.

### 3.1. JNK Dynamics in Cell Fate Determination in Cell Populations

JNK activity has long been associated with a dichotomy of regulating both cell survival and apoptosis. Initial studies first demonstrated that JNK played a critical role in the induction of neuronal apoptosis following the withdrawal of nerve growth factor (NGF) [75]. Consistent with this role, JNK1/2-deficient MEFs exhibit resistance to UV-induced apoptosis [76] and fibroblasts expressing S63A, S73A mutations within c-Jun show resistance to apoptosis [77]. This pro-apoptotic effect is not restricted to JNK1/2, as JNK3 also plays specific roles in neuronal apoptosis [18]. Similarly, JNK has known roles in driving cell death in response to TNFα [38], H_2_O_2_ [78], endoplasmic reticulum stress (ER-stress) [10] and in some instances of γ-irradiation [4,5]. In contrast, JNK activity has been shown to play key roles in development, cellular survival, proliferation, and transformation. For example, the loss of both JNK1 and JNK2 results in embryonic lethality, demonstrating a requirement for normal development [79], and the loss of JNK also promotes premature cellular senescence [80]. The JNK target c-Jun is also critical to maintain cellular proliferation by driving the expression of cyclin D1 [81], and cellular transformation via Ras requires c-Jun [82].

**Figure 2:**
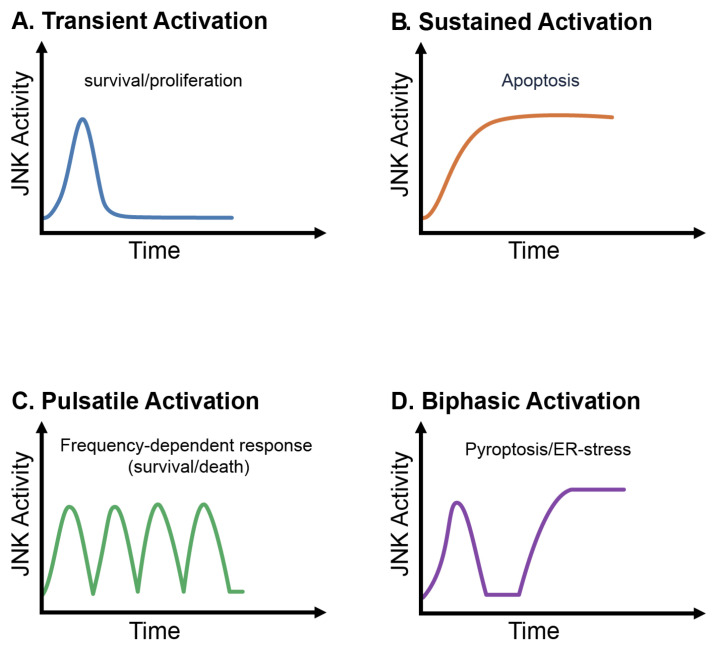
Temporal dynamics of JNK and response. JNK exhibits an array of temporal dynamics including transient (**A**), sustained (**B**), pulsatile or oscillatory (**C**), and biphasic activation (**D**). These patterns are associated with discrete cell fate outcomes including survival, proliferation, apoptosis, pyroptosis, and ER-stress responses.

Extensive work has demonstrated that these seemingly contradictory outcomes rely on the relative duration or dynamics of JNK activation over time (Figure 2). For example, prolonging JNK activation in response to sodium orthovanadate by co-treatment with PMA, and Ionomycin increased cell death in Jurkat cells [4]. Similarly, chemigenetic approaches also demonstrate the importance of sustained JNK activation in the transduction of apoptotic signals [83]. These dynamics are not limited to transient or sustained dynamics either, as biphasic patterns of JNK activation regulate cellular responses to ER stress. An early cytoprotective pulse promotes the expression of anti-apoptotic genes, which was followed by a delayed cytotoxic rise in JNK activation [84,85]. One limitation of these early studies is the reliance on immunoblots or other population level measurements [86,87] that limit the ability to track cellular heterogeneity. Additionally, the reliance on fixed time points provides only a snapshot in time. However, developments within the field of microscopy and fluorescence approaches have now provided unprecedented insight into the single cell biology of JNK signaling.

### 3.2. Live-Cell Imaging and Single-Cell Dynamics in the Regulation of Cell Fate

Over the last decade, attention has shifted from population dynamics to single-cell dynamics of signaling molecules (e.g., kinases) to evaluate their consequences in cellular responses. This trend has been accelerated and supported by the tremendous advancements in the development of new sophisticated tools and methods including biosensors for live-cell imaging, mathematical and computational modeling, optogenetic systems, and microfluidic devices [88,89,90,91]. Initially, two fluorescent protein FRET-based systems were used to characterize the signaling dynamics of kinases [92,93]; however, these approaches can be challenging to optimize and often require specialized filter sets. More recently, the development of single fluorescent protein kinase translocation reporters (KTRs) [89] has greatly accelerated the study of kinase dynamics in single cells across diverse stimuli [35,40,94,95,96]. These simple biosensors allow for complex multiplexing, increasing the range of pathways that can be imaged simultaneously.

Studies that have utilized these biosensors have not only supported prior findings but have offered additional insights into the behavior of single cells and heterogeneity in responses across cells. For example, the relative strength of negative regulation by p38 or the phosphatase DUSP1 can shape temporal JNK dynamics to a range of stimuli, including sorbitol, TNFα, anisomycin, and IL-1β [95]. Variations in the cell-to-cell expression of DUSP1 also determines whether cells exhibit sustained JNK activation and undergo UV-induced cell death [95]. In contrast, oxidative stress-induced JNK activation exhibits secondary pulses associated with cell death, while cells that survive tend to lack additional JNK activation [96]. The same JNKKTR-based approach has also shown that the levels of ZAK help regulate cell survival, tolerance, or apoptosis in response to ribotoxic stress [37]. Other single-cell dynamics that have been observed include the biphasic activation of JNK with early JNK activation tightly correlated with a mitochondrial ROS (mtROS) spike that licenses inflammasome assembly [40]. This early phase is subsequently followed by a second phase of JNK activation 2 h later driven by a MAP3K5/JNK2 signaling complex to promote GSDMD mobilization and pyroptotic cytokine release [40]. While these studies illustrate the potential applications of the JNKKTR biosensor, the growing use of computational approaches coupled with imaging are providing even further insights into the roles of JNK over time. For example, fluorescence imaging coupled with TENSOR models has defined roles for biphasic JNK signaling in the induction of senescence and regulation of the senescence-associated secretory phenotype (SASP) [97].

While the above approaches primarily focus on the relationship between JNK activation and cell fate, recent studies are using these tools to explore how the combinatorial array of MAP3K and MAP2K proteins contribute to the regulation of these signaling networks over time. A systematic analysis of MAP3Ks has shown that different MAP3Ks elicit distinct MAPK dynamics for ERK, JNK, and p38 [35]. Interestingly, these studies suggest that JNK dynamics alone do not specify cell fate. Rather, the complex combination of ERK, JNK, and p38 activation is ultimately what determines the distinction between proliferation or cell death [35]. Studies utilizing fluorescent tracking of the MAP2K MKK4 have also shown that the spatiotemporal dynamics of the MAP2K may contribute to JNK dynamics albeit without a direct measure of single-cell kinase activity [98]. We anticipate that future studies exploring the network of positive and negative regulators of JNK will provide further insight into how these proteins contribute to the dynamic patterns of JNK activation. However, it is important to note that many of our current live-cell readouts of kinase activity have specific limitations. For example, FRET-based reporters require precise calibration, often suffer from low signal-to-noise ratios, and imaging rates can be limited by hardware potentially missing very rapid or slow responses [99]. KTRs have their own set of limitations, including a reliance on cellular transport rates, sensitivity to crosstalk with other kinases, small dynamic range, and they do not distinguish between JNK isoforms [89,100]. Naturally, the overexpression of reporters can also potentially interfere with endogenous signaling. Addressing these weaknesses will be crucial to future studies of temporal signaling, and enhanced KTRs are already being developed to overcome some of these weaknesses [101].

### 3.3. JNK Dynamics in the Regulation of Gene Expression

Most prior studies of JNK activation dynamics have largely focused on cell fate regulation without a clear focus on transcriptional regulation despite the transcription factor c-Jun being the most well-characterized substrate of JNK. Dynamic studies of other transcription factors, including p53 [102,103,104,105] and NF-kB [8], have shown that temporal dynamics can have significant impacts on gene expression patterns. These patterns often rely heavily on the mRNA and protein stability of the downstream targets [8,104,105,106]. Based on these findings, it is rational to hypothesize that the dynamics of JNK activation are likely to influence the dynamic patterns of gene expression. Recent findings support this hypothesis, as research has demonstrated that the duration of JNK activation results in a dynamic phosphorylation of c-Jun over time [107]. Initially, this drives phosphorylation at the activation sites serine 63 and serine 73, promoting the release of the transcriptional repressor MBD3 and association with the activator TCF4 to promote transcriptional activity. However, as activation persists, the phosphorylation of threonine 91 and 93 rises, resulting in the displacement of TCF4 and returning c-Jun to an inactive state. Temporal dosing strategies to enrich specific JNK dynamics also support a role in the regulation of gene expression. For example, sustained, transient, or pulsed JNK activation drives distinct clusters of gene expression patterns [91]. Consistent with the prolonged activation of JNK pushing c-Jun to an inactive state, a transient cluster of gene expression was observed even with sustained JNK activation. These findings suggest that a short, pulsatile activation of JNK may be optimal for transcriptional regulation, while prolonged JNK activation drives non-transcriptional responses involved in the induction of cell death. Similar to p53 and NF-kB, computational modeling suggests that JNK-dependent gene expression patterns are heavily reliant on the mRNA stability of the target gene; however, other potential mechanisms include promoter strength or combinatorial regulation [91]. However, further study is needed to see if similar promoter decoding mechanisms exist for JNK and c-Jun mediated transcription as has been seen for p53 [108] and yeast promoters [109]. Within Table 2, we summarize our basic understanding of phenotypic outcomes related to specific JNK dynamics.

## 4. Spatial Dynamics of JNK in the Regulation of Cell Fate and Function

**Figure 3:**
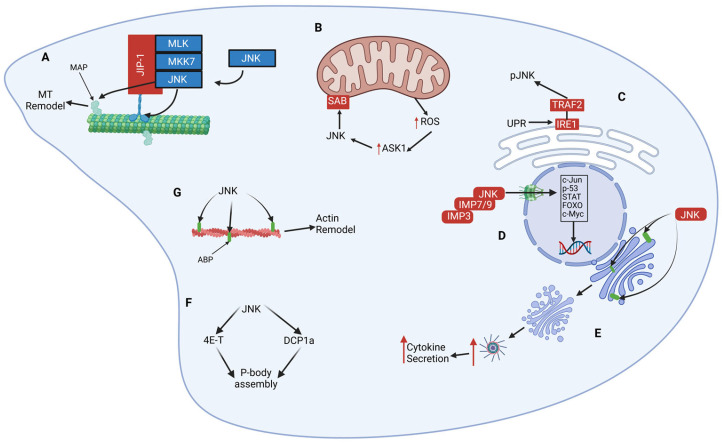
Overview of cellular JNK spatial dynamics. (**A**) JNK associates with the JIP-1 scaffold complex alongside MLK and MKK7 on the microtubule network. This localization regulates microtubule-associated proteins (MAPs) to drive microtubule (MT) remodeling. (**B**) At the outer mitochondrial membrane, JNK binds to SAB, resulting in an increased production of reactive oxygen species (ROS). Elevated ROS levels subsequently activate ASK1, establishing a positive feedback loop that sustains JNK activation. (**C**) Under stress, the unfolded protein response (UPR) triggers the IRE1–TRAF2 signaling axis, leading to the increased phosphorylation of JNK. (**D**) JNK complexes with importin proteins (IMP3 and IMP7/9) to translocate through nuclear pores into the nucleus. Once inside, it phosphorylates key transcription factors to modulate gene expression. (**E**) JNK phosphorylates certain Golgi apparatus functional proteins which can increase Golgi fragmentation. Increased fragmentation increased inflammasome formation and therefore cytokine production. (**F**) In the cytoplasm, JNK targets the RNA-binding and processing proteins 4E-T and DCP1a, driving the formation and assembly of processing bodies (P-bodies). (**G**) JNK phosphorylates actin-binding proteins (ABPs) to regulate actin filament dynamics.

The functional output of JNK signaling is determined as much by where active JNK is located as by when and how much it is activated. Spatial compartmentalization concentrates JNK with specific upstream kinases, scaffolds, substrates, and regulatory proteins to create microdomains that bias signaling toward distinct outcomes. The following section compiles the current mechanistic knowledge of JNK’s compartment-specific interactors (Figure 3), the molecular mechanisms that control JNK localization, and the downstream consequences of those interactions while also highlighting current gaps in our understanding.

### 4.1. Scaffold-Mediated Cytoplasmic Organization

Scaffold proteins are the primary architects of JNK spatial specificity, functioning as molecular platforms that sequester JNK isoforms with their respective upstream activators (MAP3Ks and MAP2Ks) and downstream targets. By organizing these components into discrete signalosomes, scaffolds ensure signal fidelity, prevent unwanted crosstalk with other MAPK pathways, and determine the specific subcellular neighborhood where JNK will phosphorylate its substrates to initiate the appropriate cellular cascades [112]. Within this section, we highlight our current understanding of the known JNK scaffolding proteins and the functional outcomes of interactions. These include the JNK-interacting protein family (JIPs), WD repeat-containing protein 62 (WDR62), Plenty of SH3 (POSH), and β-arrestins. It should be noted that while we have a strong understanding of the scaffolds that orient JNK signaling complexes, we have little understanding of how these scaffolds contribute to the temporal dynamics of JNK previously described.

#### 4.1.1. JNK-Interacting Protein Family (JIP1-4)

The JNK-interacting protein family (JIP) consists broadly of four known JNK scaffolds (JIP1-4). Structurally, each of these proteins contain a JNK-binding domain or D-domain that allows for interaction with JNK [27]. JIP1 and JIP2 are structurally similar, as each contain an additional SH3 domain that facilitates the dimerization and phosphotyrosine-binding domain to bind cargo. JIP1 is characterized by its ability to assemble a discrete signaling module consisting of JNK, MKK7, and members of the Mixed Lineage Kinase (MLK) family [113]. Beyond its structural role, JIP1 is a key regulator of neuronal development, where it facilitates the localization of JNK to the growth cones of developing axons [114,115]. It also plays a vital role in metabolic homeostasis, as it modulates the JNK response to inflammatory stimuli in insulin-sensitive cells [116].

While JIP2 shares significant sequence homology and function with JIP1, it is present in higher concentrations in the brain and nervous tissue as compared to JIP1 which is more ubiquitously expressed throughout the body [113]. In the brain, JIP2 acts as a molecular “GPS” and transport adapter that physically directs JNK signaling to specific locations in the neuron, such as synapses, to enable specialized functions like axon guidance and remodeling [117]. Another major difference between JIP1 and JIP2 is in the subcellular localization. JIP1 tends to be located more densely in the cell surface projections, whereas JIP2 is generally distributed uniformly across the cytoplasm. JIP1 also exhibits higher affinity for binding with JNK than JIP2 [113].

Unlike JIP1 and JIP2, both JIP3 and JIP4 are structurally distinct scaffolds as they lack the SH3 and PTB domains common to JIP1 and 2 [27]. The expression of JIP3 is highly enriched in the central nervous system. It serves as a specialized adapter that links the JNK (primarily JNK3) signaling module directly to the kinesin-1 motor complex. JIP3 acts as a bridge that links the kinesin-1 motor to JNK. JIP3 binding to kinesin light chains (KLCs) engages the motor with microtubules, while its binding to kinesin heavy chains (KHCs) directly triggers the motor’s motility and speed. The result of this interaction is the coordinated transport of JNK signaling cargo to the ends of nerve cells, which is essential for axon elongation during development and axon regeneration following an injury [118]. Additionally, JIP3 localizes to discrete exocytic vesicles and may recruit JNK to its substrate paxillin to facilitate neurite outgrowth [119].

JIP4 is encoded by the *SPAG9* gene, which also encodes the other JNK scaffold variants JLP (JNK-associated Leucine Zipper Protein) and SPAG9 (sperm-associated antigen 9) [120,121,122]. Like JIP3, JIP4 binds JNK and kinesin-1, but it differs significantly from JIP3 in overall function, as JIP4 specifically activates the p38 MAPK pathway rather than JNK despite having similar structural domains [120]. Instead of acting as a scaffold, JIP4 serves as a substrate, being actively phosphorylated by JNK. Unlike other JIPs, which localize JNK to activate it in specific signaling modules, JIP4 localizes JNK without activating it, suggesting it might act as a storage site or a transport vehicle rather than a signaling hub for JNK [120]. As all four of these JIP proteins rely on a JNK-binding domain to associate with JNK, the overexpression of this domain can be used to inhibit JNK activation by competing for JNK binding with active scaffold complexes [123].

#### 4.1.2. WD Repeat Domain 62 (WDR62)

WDR62 is a scaffold protein that specifically associates with JNK but not with ERK and p38 [124], and mutations in WDR62 have been associated with microcephaly [125]. During mitosis, WDR62 localizes to the centrosomes and the spindle poles where it serves as a physical platform to recruit JNK and its upstream activator, MKK7 [126,127]. This WDR62/JNK1 interaction is required for timely mitotic progression, and disruption either through the loss of WDR62, inhibition of JNK, or loss of specific JNK phosphorylation on WDR62 disrupts this process [126,128]. This disruption may be the result of altered phosphorylation kinetics of the JNK substrates Cdc25C and Ch1, both of which help coordinate cell cycle progression [129,130]. In contrast to the known roles of WDR62 in mitotic progression, it has also been shown to coordinate the recruitment of JNK to stress granules and P-bodies during the induction of cellular stress in response to an array of stimuli [124]. Interestingly, while WDR62 promotes JNK activation, it does not facilitate AP-1 dependent transcription, potentially coupling JNK function to post-transcriptional regulation [124]. Additional evidence suggests that JNK activity promotes stress granule formation, but the role of JNK in stress granules and P-bodies remains relatively understudied [131].

#### 4.1.3. Plenty of SH3 (POSH and POSH2)

Both POSH and POSH2 are large scaffold proteins characterized by the presence of multiple SH3 domains and an integrated RING finger domain that confers E3 ubiquitin ligase activity [132,133]. Despite structural similarities, POSH and POSH2 carry out distinct molecular functions. POSH interacts with JIPs to form the POSH–JIP apoptotic complex (PJAC) to promote apoptosis in association with Rac1, MAP3Ks (MLK), MAP2Ks (MKK4/7), and JNK [134]. In addition to playing a key role in apoptosis, POSH also facilitates neuronal migration through an association with Rac1 and localization to the Proximal Cytoplasmic Dilation of the Leading Process (PCDLC) of migrating neurons, potentially ensuring that JNK is at the correct location to promote migration [135]. POSH’s ubiquitin ligase activity also distinguishes it from other scaffolds, allowing for additional protein control by modulating protein stability. While JNK is not a target of POSH-mediated ubiquitination, POSH can regulate the stability of itself and other cellular pathways [132,136].

In contrast to POSH, POSH2 (or POSHER) appears to promote cell survival by regulating POSH expression in unstressed conditions [137]. However, this is likely context dependent, as the overexpression of POSH2 can still induce JNK-dependent apoptosis in specific cell lines models [138]. POSH2 also shows binding to both inactive and active Rac1, further differentiating it from POSH and suggesting that these proteins are not simply functionally redundant [137].

#### 4.1.4. β-Arrestin-2

In contrast to the previously discussed scaffolds, β-arrestin-2 is unique as a multifunctional adapter that traditionally facilitates the desensitization and endocytosis of G-protein coupled receptors (GPCRs) [139]. However, yeast two-hybrid screens identify β-arrestin-2 as specific interacting partner with the JNK3 isoform [140] unlike other scaffolds which bind multiple JNK isoforms. Like other scaffolds, β-arrestin-2 can orient the JNK3 signaling cascade through association with the MAP3K, ASK1, and MKK4 [140,141]. Under basal conditions, β-arrestin-2 actively sequesters JNK outside the nucleus to prevent unwanted transcription [142]. While the functional role of β-arrestin-2/JNK3-mediated signaling is still being explored, recent studies suggest potential importance in dopamine-mediated behavioral responses and in pancreatic islet cell growth [143,144].

### 4.2. JNK Interactions with Cytoskeletal Proteins

The structural integrity and dynamic versatility of the cell are maintained by the cytoskeleton, which is a complex network of protein filaments that JNK modulates to coordinate morphological changes. By phosphorylating specific cytoskeletal components and their regulatory proteins, JNK serves as a bridge between extracellular stress signals and physical cellular remodeling.

#### 4.2.1. Actin

Actin filaments are semi-flexible, polar polymers that form the primary machinery for cell shape maintenance, cytokinesis, and the generation of contractile forces. JNK does not directly phosphorylate actin but rather regulates actin and cytoskeletal structures through the association with actin binding proteins (ABP), such as MARCKS-like protein-1, cofilin-1, CapZIP, SMTL2, paxillin, and the β-catenin/α-catenin complex [145,146,147,148,149,150]. The interaction of JNK with these proteins can have profound impacts on cytoskeletal function. For example, the phosphorylation of paxillin promotes cellular migration [150] and the phosphorylation of β-catenin promotes the dissociation of cell-to-cell contacts and adherens junctions [148]. More recently, JNK has been shown to regulate the assembly of cilia through its action in actin regulation [151].

#### 4.2.2. Microtubules (MTs)

Microtubules are stiff, hollow tubes composed of α-and β-tubulin dimers that function as a structural scaffold and transport system for intracellular organelles and vesicles. JNK and microtubules have a bidirectional signaling relationship, as microtubule dynamics can control JNK activation and JNK can directly influence microtubule dynamics. Since JIPs act as cargo of kinesins, microtubules are a key part of JNK spatial regulation [152]. Cell starvation can lead to the hyperacetylation of MTs which can then recruit and stimulate JNK via the increased binding of additional JIP-bearing kinesins to MTs [153]. Other types of cell stress, such as oxidative stress, have also been shown to result in different JNK-mediated microtubule effects [154]. JNK indirectly regulates MTs by phosphorylating microtubule-associated proteins (MAPs). JNK is known to phosphorylate MAP1B, MAP2, and Tau to increase neuronal growth and stability [155,156]. JNK also directly phosphorylates kinesin motors (KIF5C) [157] and their cargoes (synaptotagmin-4 [158], Bim, and Bmf [65]) to regulate MT-based transport. In the case of Bim and Bmf, this phosphorylation is critical for release from motor complexes and the induction of apoptosis.

#### 4.2.3. Intermediate Filaments (IFs)

Intermediate filaments provide the cell with mechanical durability and stress resistance, acting as a tensile scaffold that anchors organelles and maintains the structural integrity of the nuclear envelope. IFs, like MTs, have a bidirectional relationship with JNK. IFs like desmin, vimentin and keratin-8 (K8) act as scaffolding proteins that bind to JNK to control its spatial localization and substrate availability. Desmin and vimentin increase JNK (specifically JNK2) activity [159], while K8 has the opposite effect and may sequester JNK from its substrate c-Jun [160]. This recruitment of JNK to K8 results in the phosphorylation of K8 in response to both Fas stimulation and in response to the transglutaminase-2 binding of JNK [160,161]. This phosphorylation of K8 can promote perinuclear reorganization and cell migration in cancer cells [161]. Other IFs phosphorylated via JNK included both heavy and medium neurofilaments (NFH and NFM) as well as the nuclear IF Lamin B1 [162,163].

### 4.3. Organelle- and Synapse-Specific JNK Localization

Not only can JNK mediate cytoskeletal remodeling and transport, JNK can also be localized to an array of cellular organelles and structures. Subcellular organelles act as specialized signaling hubs that allow the cell to compartmentalize JNK activity, ensuring that stress responses are specific to the localized damage. By anchoring JNK to specific organelle membranes, the cell can fine-tune its metabolism, protein folding, and communication pathways before committing to a global response like apoptosis. Within this section, we focus upon the localization of JNK to non-nuclear organelles and cellular structures independent of its known nuclear localization and function, which will be discussed within the next section.

#### 4.3.1. Mitochondria

Mitochondria are the primary sites of ATP production and are central regulators of the intrinsic apoptotic pathway driven by the release of cytochrome c from the intermembrane space [164]. In response to severe cellular stress, JNK translocates to the outer mitochondrial membrane via the membrane-bound scaffold protein SAB (SH3BP5), which is the only identifiable docking site for JNK on the outer mitochondrial membrane (OMM) [165]. This interaction typically signals irreversible cell death. When phosphorylated JNK binds to the kinase interaction motif (KIM) of SAB, this triggers a signaling cascade that results in dysregulation of the electron transport chain (ETC) and an increase in ROS production [10]. These ROS diffuse into the cytosol and activate the upstream MAP3K ASK1, creating a self-sustaining loop that maintains and amplifies JNK activity [166]. As previously highlighted, JNK can also potentiate the induction of apoptosis by phosphorylating or regulating the localization of many pro-apoptotic factors [65,71,167]. Critically, blocking this interaction between JNK and Sab has been shown to represent a potential therapeutic approach to disrupt JNK-mediated apoptosis [168,169]. Outside of its role in apoptosis, JNK can also regulate both mitochondrial fission and mitophagy through the phosphorylation of Drp1 and BNIP3 [63,64].

#### 4.3.2. Endoplasmic Reticulum (ER)

The endoplasmic reticulum is responsible for protein synthesis, folding, and calcium homeostasis. When the ER is stressed, which is generally caused by an accumulation of misfolded proteins, a condition known as the Unfolded Protein Response (UPR) occurs. The UPR includes the activation of JNK by the ER transmembrane protein IRE1α and the adaptor TRAF2 [170]. Persistent JNK activation at the ER eventually leads to the depletion of calcium stores and the activation of caspases, linking ER stress to cellular death via JNK signaling [171]. This process can be dynamic; as discussed earlier, ER stress can induce two phases of JNK activation with the early activation, favoring the expression of pro-survival genes before ultimately driving cell death at later phases [84]. Interestingly, mitochondria-ER contact sites (MERCS) have been shown to be critical signaling nodes to regulate crosstalk between ER and mitochondrial function and apoptosis; however, there has been no direct link shown between JNK and MERCs to date [172]. Given the importance of these organelle–organelle contacts, future research to define JNK’s potential roles is critical.

#### 4.3.3. Golgi Apparatus

The Golgi apparatus serves as the central sorting and dispatching station for proteins and lipids within the secretory pathway. When triggered by inflammatory signals (like IL-1β) or when treated with anisomycin, JNK can phosphorylate numerous proteins (GRASP55, GRASP65, CAMSAP2, TJAP1, YIPF2) associated with Golgi apparatus function and assembly [173]. This results in the formation of an increasingly fragmented Golgi which promotes increased secretion rates of cytokines during stress. Given the recent implications of JNK in inflammasome activation and GASDMD pore formation, this positions JNK as a critical regulator of cytokine release [40]. Other Golgi-bound proteins like AKRL1/2 are able to increase JNK activity by promoting signal transduction through the MAP3K TAK1 and both MKK4 and MKK7 [174]. The mechanistic implications of this potential signaling network is currently unclear, though in certain studies, AKRL1/2 have been associated with Huntington’s disease [175], which would be consistent with JNK’s significant functions in regulating neural development and function.

#### 4.3.4. Synapse

JNK is localized to the synapse through its association with several scaffold proteins that have already been discussed such as JIP, β-arrestin-2 and POSH. These scaffold proteins organize JNK and its activators into signalosomes and facilitate its delivery to and from synapses, where it is usually carried by kinesins on microtubules. The synapse itself serves as a critical regulator of JNK function. For example, the stimulation of postsynaptic NMDA receptors (NMDARs) acts as a powerful activator of the JNK signaling cascade. When JNK is activated by pre-synaptic NMDARs, it acts as a physiological effector and interacts with the T-SNARE proteins Syntaxin-1, Syntaxin-2, and SNAP-25 to facilitate neurotransmitter release [176]. At the postsynaptic density (PSD), the amount of JNK present directly controls the amount of key receptor and scaffold proteins, such as GluN2A, GluN2B, GluA1, GluA2, PSD-95 and drebrin; this helps to control the amount of neurotransmitter received [177].

#### 4.3.5. Stress Granules and P-Bodies

As previously indicated, JNK can localize to both stress granules and P-bodies, which play a crucial role in the regulation of mRNA and translation [178]. This localization relies on the previously discussed WDR62 scaffold [124]. However, the precise role of JNK within these membraneless organelles remains unclear. JNK-mediated phosphorylation of the eIF4E-binding protein 4E-T can promote P-body assembly, but it does not appear to alter protein synthesis rates [179]. Phosphorylation of the decapping enzyme DCP1α is also important for P-body formation and the localization of DCP1α, but it does not appear to influence its enzymatic activity [180]. However, mutant DCP1α results in an altered expression of IL-1-induced genes, suggesting potential mRNA stability regulation via JNK-mediated P-bodies [180]. As such, the further exploration of JNKs role in stress granule and P-body function is needed to understand the mechanistic role of JNK in regulating the function of these structures.

### 4.4. Nuclear Translocation and Genetic Targets

The translocation of JNK into the nucleus is a highly orchestrated transition that begins with the kinase’s release from cytoplasmic sequestration, which is the default location of JNK under normal conditions. JNK is often tethered to scaffold proteins such as JIP1, which keep the signaling module localized to specific cytoplasmic neighborhoods to prevent premature activation. Because JNK lacks a classical nuclear localization signal, it relies on specific importins to facilitate its movement through the nuclear envelope. Upon exposure to environmental stress or growth factors, JNK forms a heterotrimer with the importin proteins Imp7/9 and Imp3 [181]. This complex carries JNK to the nuclear envelope where it passes through the nuclear pores. IMP7/9 carry JNK through the pore where Ran further dissociates the complex, freeing JNK within the nucleus.

Once inside the nucleus, JNK acts as a master epigenetic and transcriptional architect by physically modifying the Transactivation Domain (TAD) of essential transcription factors. The most prominent target is c-Jun, which JNK phosphorylates to enhance its ability to form the AP-1 complex and initiate gene transcription. The biological breadth of nuclear JNK signaling is achieved through its interaction with a diverse array of transcription factors beyond the AP-1 family, including ATF2, Elk1, p53, and FOXO proteins [66,74,182]. JNK’s ability to phosphorylate these various targets allows it to function as a molecular switch that can drive vastly different pathways depending on the cellular context and the specific assembly of transcription complexes.

## 5. Clinical Relevance of JNK as a Therapeutic Target

As we have highlighted within this review, JNK plays critical roles in an array of cellular functions including cell survival, death, and inflammatory response. Not surprisingly, JNK has been an attractive therapeutic target based on these cellular functions in both cancer and inflammatory disease. Numerous kinase inhibitors have been developed to target JNK activation, including ATP-competitive inhibitors and covalent inhibitors [183]. However, clinical efficacy has been elusive for the direct inhibition of JNK activity. For example, the inhibitor CC-930 was evaluated in clinical trials of pulmonary fibrosis and ultimately discontinued due to adverse toxicity [184]. More recent trials using more advanced inhibitors are showing potential promise, as the peptide inhibitor brimapitide shows efficacy in preventing hearing loss [185] and CC-90001 exhibits decreased adverse effects as a treatment for pulmonary fibrosis [186]. However, at present, there remain no FDA-approved JNK inhibitors. In contrast to the global inhibition of JNK activity, the disruption of JNK’s interaction with specific partners may represent the best approach to minimize the toxicity associated with JNK inhibition. Preclinical studies using the JNK-Sab inhibiting peptide Tat-SabKIM1 has shown improvements in rat models of ischemia and 6-hydroxydopamine models of Parkinson’s disease [168,187]. However, this peptide has not been evaluated in clinical trials to date. Other strategies to target JNK include targeting downstream transcription via AP-1. T-5224, a small molecule inhibitor of AP-1, limited arthritis by inhibiting matrix metalloproteinase production, but clinical trials were ultimately discontinued [188]. As such, there is a remaining need to better understand the links between JNK activation, localization, and function to better target it in the clinic.

## 6. Discussion: Gaps and Opportunities

Within this review, we have highlighted our current understanding of the biochemical function and regulation of JNK through both temporal and spatial mechanisms. The timing of JNK activation plays precise roles in the determination of cell fate in response to an array of stimuli. Furthermore, where JNK is located upon activation further specifies the cellular response by localizing to mitochondria to induce apoptosis, inducing gene expression through nuclear localization, or remodeling cytoskeletal structures to adapt to stimulation. Ultimately, JNK is a critical spatiotemporal regulator of both cell fate and function. However, despite extensive study, there are many remaining gaps in our understanding of how the spatial and temporal activation of JNK coordinate to contribute to its diverse functions within a cell. With the continued growth of single-cell and live cell-based approaches, we anticipate that continued studies exploring these areas will offer additional insight into the function of this critical signaling pathway. In this section, we highlight two specific areas of potential future research exploration.

### 6.1. Dynamic Regulation of Transcription Beyond Jun

While c-Jun is the most well-known and characterized of JNKs substrates, proteomic studies have identified numerous transcription factors as substrates of JNK. Included among these are major transcriptional regulators such as c-Myc [68], p53 [189], and the STAT [73] family of transcription factors. In the case of p53, JNK has been shown to phosphorylate p53 at S6, T81, and potentially S33 in human cells [67,189,190] and S34 in mouse cells [182], often in response to UV-associated stress to potentiate apoptosis; however, recent studies also implicate JNK and p53 crosstalk in mediating cell death during replication catastrophe [191]. While these phosphorylation sites appear to increase the stability of p53 and promote transcriptional activity, the interplay of JNK and p53 may be complex and context dependent. Evidence suggests that inactive JNK can promote p53 degradation in unstressed cells [192], and the loss of JNK can promote premature senescence via the upregulation of p53 [80]. Similarly, c-Jun can transcriptionally repress the expression of p53 [193], suggesting that JNK can regulate both the accumulation and repression of p53 through multiple mechanisms. Interestingly, p53 itself may influence the dynamics of JNK activity, as the binding of p53 prevents the dephosphorylation of JNK via MKP5 (DUSP10) [194]. Given that p53 has been shown to exhibit an array of temporal dynamics in response to a variety of stimuli [195,196,197], mediating both cell fate [6,7,198] and transcriptional responses over time [96,102,104,105,199], it is likely that these two critical stress-responsive pathways play an important role in the temporal coordination of cellular stress and repair. However, our current understanding of the temporal crosstalk between these pathways is relatively limited. In response to oxidative stress, it was shown that the dynamics of p53, JNK, and p38 ultimately play a role in mediating cell death or survival [96], but further exploration of the temporal crosstalk is likely needed across multiple stimuli.

Beyond p53, JNK activity is also highly integrated with NF-kB signaling, albeit more indirectly, as NF-kB opposes JNK-mediated cell death via the transcriptional regulation of anti-apoptotic proteins like XIAP [200,201]. Like p53, NF-kB is a highly dynamic transcription factor whose dynamics play a key role in mediating inflammatory responses [202,203]. The iterative modeling of gene regulatory networks in response to a variety of inflammatory signals suggests potential transcriptional crosstalk between AP-1, NF-kB, and p38 regulated gene expression, further highlighting the need for multifactorial temporal studies [204]. Even transcriptional regulation via c-Jun/AP-1 alone may be more temporally complex than initially thought. Within mammalian cells, the 53 bZIP proteins, like c-Jun, can potentially engage in 1431 distinct dimer combinations, and in many cases, these dimers exhibit distinct DNA-binding capacity [205,206]. Given that Jun and Fos family proteins, the most well-known components of AP-1, can exhibit varying expression patterns over time [207,208], it is possible that the prolonged activation of JNK may drive distinct transcriptional profiles over time. As such, the continued development of fluorescent reporters, CRISPR-mediated tagging, and advances in live cell imaging is likely to provide further insight into the complex transcriptional network associated with JNK activity.

### 6.2. Dissecting the Relationship of Spatial and Temporal JNK Activity

Throughout this review, we highlight that both the spatial location and temporal dynamics of JNK play a critical role in mediating cellular responses ranging from gene expression to cell fate, cytoskeletal organization, and intracellular transport. At the organismal level, the localization and activity of JNK can have significant impacts on growth and development. For example, JNK signaling within the *Drosophila* regulates wing growth [209] and wound healing in a localization-dependent manner [210]. Similarly, recent studies using the JNKKTR have shown differences in spatial JNK activity within the developing spinal cord of zebrafish and a role in glial cell cycling during injury [211]. However, at the cellular level, we have less understanding of how the spatial and temporal patterns of JNK relate to one another. FRET-based sensors have provided some hints that the spatial activity of JNK may change over time [93]: FRET sensors tethered to mitochondria, the plasma membrane, or localized to the nucleus have shown that upon anisomycin treatment, JNK was active within all three compartments. However, activation at mitochondria and the plasma membrane may precede nuclear activation albeit with relatively modest changes with peak FRET emissions for mitochondria at ~20 min and peak nuclear signal at ~80 min [93]. Some limitations of these studies are that they lack the deep temporal information acquired, with recent JNKKTR studies spanning hours to days, and utilize a single stimulus, which may limit our ability to track prolonged changes to JNK activity over time or in response to different stimuli. Additional evidence also suggests that Sab is critical for the maintenance of sustained JNK activation associated with the induction of cell death [10]. However, these studies lack the fine temporal and spatial resolution to define a direct relationship. The development of novel optogenetic tools to precisely manipulate both protein localization and activation in a precise spatial and temporal manner via light may provide novel approaches to dissecting these relationships [212]. Additionally, proximity-based proteomics tools like BioID and APEX2 should provide further insight into not only specific JNK substrates but also the spatial identity of these substrates through time [213,214].

## 7. Conclusions

JNK is a master regulator of cell fate, gene expression, and cellular function across biological systems. In order to tightly coordinate appropriate cellular responses to stress stimuli, JNK activity is regulated through both space and time. Despite extensive research over multiple decades, many knowledge gaps remain in how individual cells ultimately regulate cell fate determination and how spatial localization and temporal dynamics coordinate to drive these effects. We envision that future research utilizing advanced single-cell approaches will provide insight into how the diverse array of scaffolds, phosphatases, and substrates ultimately shape the dynamic response of JNK through space and time.

## Figures and Tables

**Table 1:** Summary of JNK gene and isoform differentiation.

JNK Isoform	Location	Functions	Citations
JNK1	Ubiquitously expressed throughout most tissues	Lower c-Jun affinity than JNK2; JNK1 loss reduces liver injury, reduces fibroblast growth, decreases epidermis cell layers; JNK1 activity negatively regulates Th2 differentiation	[13,14,16,19,20,21,22,23]
JNK2	Ubiquitously expressed throughout most tissues	Higher affinity for c-Jun than JNK1; JNK2 loss increases liver injury, increases fibroblast growth, and drives keratinocyte hyperplasia; JNK2 activity is required for Th1 differentiation	[14,19,20,22,24]
JNK3	Brain, heart, testes, and pancreas	Neuronal apoptosis and inflammation, regulates β-cell function, regulates ischemia-induced brain injury	[15,17,23,25]

**Table 2:** Summary of observed JNK dynamics and phenotypes.

Dynamics	Duration	Phenotypic Outcomes (Cell Fate)	References
Transient Activation	Minutes to a few hours	Cell survival and proliferation	[91,95,110]
Sustained Activation	Extended, unattenuated plateau lasting 4 to 12+ h.	Apoptosis	[91,97,111]
Pulsatile/Oscillatory	Intermittent bursts recurring over several hours.	Frequency dependent: survival, cell death, gene regulation	[91,95]
Biphasic Dynamics	Two distinct waves (Wave 1: ~15–30 m; Wave 2: delayed 2–6 h later).	Pyroptosis, ER-stress response, senescence regulation	[40,84,97]

## Data Availability

No new data were created or analyzed in this study. Data sharing is not applicable to this article.

## Abbreviations

The following abbreviations are used in this manuscript:

ABP	Actin-binding protein
ASK1	Apoptosis-stimulating kinase 1
BNIP3	BCL2 interacting protein 3
DLK	Dual leucine zipper kinase
Drp-1	Dynamin-related protein 1
DUSP	Dual specificity phosphatase
ER-stress	Endoplasmic reticulum stress
ERK	Extracellular signal-regulated kinase
FOXO	Forkhead box O
FRET	Förster resonance energy transfer
GSDMD	Gasdermin D
GTSP	Glutathione S-Transferase Pi
IF	Intermediate filament
IL-1β	Interleukin-1 beta
IMP	Importin
JNK	c-Jun N-terminal kinase
JIP	JNK-interacting protein
K8	Keratin-8
KIF5C	Kinesin family member 5C
KTR	Kinase translocation reporter
LZK	Leucine zipper kinase
MAP	Microtubule-associated protein
MAPK	Mitogen-activated protein kinase
MAP2K	MAP kinase kinase
MAP3K	MAP kinase kinase kinase
MBD3	Methyl-CpG-binding domain protein 3
MEF	Mouse embryonic fibroblast
MERCS	Mitochondria-ER contact sites
MKK3	Mitogen-activated protein kinase kinase 3
MKK4	Mitogen-activated protein kinase kinase 4
MKK7	Mitogen-activated protein kinase kinase 7
MKP	MAPK phosphatase
MLK	Mixed lineage kinase
MT	Microtubule
mtROS	Mitochondrial ROS
NFH	Heavy neurofilament
NFM	Medium neurofilament
NGF	Nerve growth factor
NMDAR	NMDA receptor
OMM	Outer mitochondrial membrane
PMA	Phorbol 12-myristate 13-acetate
POSH	Plenty of SH3
PSD	Postsynaptic density
ROS	Reactive oxygen species
SAB	SH3 domain-binding protein 5 (SH3BP5)
SAPK	Stress-activated protein kinase
SASP	Senescence-associated secretory phenotype
SRFP	Secreted frizzled-related protein
STAT	Signal transducer and activator of transcription
TAB1	TAK1-binding protein 1
TAD	Transactivation Domain
TCF4	Transcription factor 4
TNFα	Tumor necrosis factor alpha
TRAF2	TNF receptor-associated factor 2
Trx-1	Thioredoxin-1
UPR	Unfolded protein response
UV	Ultraviolet
WDR62	WD repeat-containing protein 62
ZAK	Sterile alpha motif and leucine zipper containing kinase

## References

[1] Kyriakis J.M., Avruch J. (1990). pp54 microtubule-associated protein 2 kinase. A novel serine/threonine protein kinase regulated by phosphorylation and stimulated by poly-L-lysine. J. Biol. Chem..

[2] Kyriakis J.M., Avruch J. (2001). Mammalian Mitogen-Activated Protein Kinase Signal Transduction Pathways Activated by Stress and Inflammation. Physiol. Rev..

[3] Weston C.R., Davis R.J. (2002). The JNK signal transduction pathway. Curr. Opin. Genet. Dev..

[4] Chen Y.R., Wang X., Templeton D., Davis R.J., Tan T.H. (1996). The role of c-Jun N-terminal kinase (JNK) in apoptosis induced by ultraviolet C and gamma radiation. Duration of JNK activation may determine cell death and proliferation. J. Biol. Chem..

[5] Chen Y.R., Meyer C.F., Tan T.H. (1996). Persistent activation of c-Jun N-terminal kinase 1 (JNK1) in gamma radiation-induced apoptosis. J. Biol. Chem..

[6] Paek A.L., Liu J.C., Loewer A., Forrester W.C., Lahav G. (2016). Cell-to-Cell Variation in p53 Dynamics Leads to Fractional Killing. Cell.

[7] Purvis J.E., Karhohs K.W., Mock C., Batchelor E., Loewer A., Lahav G. (2012). p53 dynamics control cell fate. Science.

[8] Zambrano S., De Toma I., Piffer A., Bianchi M.E., Agresti A. (2016). NF-kappaB oscillations translate into functionally related patterns of gene expression. eLife.

[9] Lasick K.A., Jose E., Samayoa A.M., Shanks L., Pond K.W., Thorne C.A., Paek A.L. (2023). FOXO nuclear shuttling dynamics are stimulus-dependent and correspond with cell fate. Mol. Biol. Cell.

[10] Win S., Than T.A., Fernandez-Checa J.C., Kaplowitz N. (2014). JNK interaction with Sab mediates ER stress induced inhibition of mitochondrial respiration and cell death. Cell Death Dis..

[11] Davis R.J. (2000). Signal transduction by the JNK group of MAP kinases. Cell.

[12] Caunt C.J., Keyse S.M. (2013). Dual-specificity MAP kinase phosphatases (MKPs): Shaping the outcome of MAP kinase signalling. FEBS J..

[13] Dérijard B., Hibi M., Wu I.H., Barrett T., Su B., Deng T., Karin M., Davis R.J. (1994). JNK1: A protein kinase stimulated by UV light and Ha-Ras that binds and phosphorylates the c-Jun activation domain. Cell.

[14] Kallunki T., Su B., Tsigelny I., Sluss H.K., Dérijard B., Moore G., Davis R., Karin M. (1994). JNK2 contains a specificity-determining region responsible for efficient c-Jun binding and phosphorylation. Genes. Dev..

[15] Mohit A.A., Martin J.H., Miller C.A. (1995). p493F12 kinase: A novel MAP kinase expressed in a subset of neurons in the human nervous system. Neuron.

[16] Sabapathy K., Hochedlinger K., Nam S.Y., Bauer A., Karin M., Wagner E.F. (2004). Distinct roles for JNK1 and JNK2 in regulating JNK activity and c-Jun-dependent cell proliferation. Mol. Cell.

[17] Keramaris E., Vanderluit J.L., Bahadori M., Mousavi K., Davis R.J., Flavell R., Slack R.S., Park D.S. (2005). c-Jun N-terminal Kinase 3 Deficiency Protects Neurons from Axotomy-induced Death in Vivo through Mechanisms Independent of c-Jun Phosphorylation*. J. Biol. Chem..

[18] Yang D.D., Kuan C.Y., Whitmarsh A.J., Rincón M., Zheng T.S., Davis R.J., Rakic P., Flavell R.A. (1997). Absence of excitotoxicity-induced apoptosis in the hippocampus of mice lacking the Jnk3 gene. Nature.

[19] Singh R., Wang Y., Xiang Y., Tanaka K.E., Gaarde W.A., Czaja M.J. (2009). Differential effects of JNK1 and JNK2 inhibition on murine steatohepatitis and insulin resistance. Hepatology.

[20] Sabapathy K., Kallunki T., David J.P., Graef I., Karin M., Wagner E.F. (2001). c-Jun NH2-terminal kinase (JNK)1 and JNK2 have similar and stage-dependent roles in regulating T cell apoptosis and proliferation. J. Exp. Med..

[21] Constant S.L., Dong C., Yang D.D., Wysk M., Davis R.J., Flavell R.A. (2000). JNK1 is required for T cell-mediated immunity against Leishmania major infection. J. Immunol..

[22] Weston C.R., Wong A., Hall J.P., Goad M.E., Flavell R.A., Davis R.J. (2004). The c-Jun NH2-terminal kinase is essential for epidermal growth factor expression during epidermal morphogenesis. Proc. Natl. Acad. Sci. USA.

[23] Dong C., Yang D.D., Wysk M., Whitmarsh A.J., Davis R.J., Flavell R.A. (1998). Defective T cell differentiation in the absence of Jnk1. Science.

[24] Yang D.D., Conze D., Whitmarsh A.J., Barrett T., Davis R.J., Rincón M., Flavell R.A. (1998). Differentiation of CD4+ T cells to Th1 cells requires MAP kinase JNK2. Immunity.

[25] Kuan C.-Y., Whitmarsh A.J., Yang D.D., Liao G., Schloemer A.J., Dong C., Bao J., Banasiak K.J., Haddad G.G., Flavell R.A. (2003). A critical role of neural-specific JNK3 for ischemic apoptosis. Proc. Natl. Acad. Sci. USA.

[26] Widmann C., Gibson S., Jarpe M.B., Johnson G.L. (1999). Mitogen-activated protein kinase: Conservation of a three-kinase module from yeast to human. Physiol. Rev..

[27] Dhanasekaran D.N., Kashef K., Lee C.M., Xu H., Reddy E.P. (2007). Scaffold proteins of MAP-kinase modules. Oncogene.

[28] Noguchi T., Takeda K., Matsuzawa A., Saegusa K., Nakano H., Gohda J., Inoue J.-I., Ichijo H. (2005). Recruitment of Tumor Necrosis Factor Receptor-associated Factor Family Proteins to Apoptosis Signal-regulating Kinase 1 Signalosome Is Essential for Oxidative Stress-induced Cell Death*. J. Biol. Chem..

[29] Weijman J.F., Kumar A., Jamieson S.A., King C.M., Caradoc-Davies T.T., Ledgerwood E.C., Murphy J.M., Mace P.D. (2017). Structural basis of autoregulatory scaffolding by apoptosis signal-regulating kinase 1. Proc. Natl. Acad. Sci. USA.

[30] Zhang H., Gallo K.A. (2001). Autoinhibition of Mixed Lineage Kinase 3 through Its Src Homology 3 Domain*. J. Biol. Chem..

[31] Cheng J., Yu L., Zhang D., Huang Q., Spencer D., Su B. (2005). Dimerization through the catalytic domain is essential for MEKK2 activation. J. Biol. Chem..

[32] Kutuzov M.A., Andreeva A.V., Voyno-Yasenetskaya T.A. (2005). Regulation of Apoptosis Signal-regulating Kinase 1 (ASK1) by PolyamineLevels via Protein Phosphatase5*. J. Biol. Chem..

[33] Raman M., Chen W., Cobb M.H. (2007). Differential regulation and properties of MAPKs. Oncogene.

[34] Chen W., White M.A., Cobb M.H. (2002). Stimulus-specific requirements for MAP3 kinases in activating the JNK pathway. J. Biol. Chem..

[35] Peterson A.F., Ingram K., Huang E.J., Parksong J., McKenney C., Bever G.S., Regot S. (2022). Systematic analysis of the MAPK signaling network reveals MAP3K-driven control of cell fate. Cell Syst..

[36] Huso V.L., Niu S., Catipovic M.A., Saba J.A., Denk T., Park E., Cheng J., Berninghausen O., Becker T., Green R. (2026). ZAK activation at the collided ribosome. Nature.

[37] Sinha N.K., McKenney C., Yeow Z.Y., Li J.J., Nam K.H., Yaron-Barir T.M., Johnson J.L., Huntsman E.M., Cantley L.C., Ordureau A. (2024). The ribotoxic stress response drives UV-mediated cell death. Cell.

[38] Deng Y., Ren X., Yang L., Lin Y., Wu X. (2003). A JNK-Dependent Pathway Is Required for TNFα-Induced Apoptosis. Cell.

[39] Reinhard C., Shamoon B., Shyamala V., Williams L.T. (1997). Tumor necrosis factor alpha-induced activation of c-jun N-terminal kinase is mediated by TRAF2. Embo J..

[40] Bradfield C.J., Liang J.J., Ernst O., John S.P., Sun J., Ganesan S., de Jesus A.A., Bryant C.E., Goldbach-Mansky R., Fraser I.D.C. (2023). Biphasic JNK signaling reveals distinct MAP3K complexes licensing inflammasome formation and pyroptosis. Cell Death Differ..

[41] Lawler S., Fleming Y., Goedert M., Cohen P. (1998). Synergistic activation of SAPK1/JNK1 by two MAP kinase kinases in vitro. Curr. Biol..

[42] Deacon K., Blank J.L. (1997). Characterization of the Mitogen-activated Protein Kinase Kinase 4 (MKK4)/c-Jun NH2-terminal kinase 1 and MKK3/p38 Pathways Regulated by MEK Kinases 2 and 3: MEK KINASE 3 ACTIVATES MKK3 BUT DOES NOT CAUSE ACTIVATION OF p38 KINASE IN VIVO*. J. Biol. Chem..

[43] Caliz A.D., Yoo H.J., Vertii A., Dolan A.C., Tournier C., Davis R.J., Keaney J.F., Kant S. (2021). Mitogen Kinase Kinase (MKK7) Controls Cytokine Production In Vitro and In Vivo in Mice. Int. J. Mol. Sci..

[44] Tournier C., Dong C., Turner T.K., Jones S.N., Flavell R.A., Davis R.J. (2001). MKK7 is an essential component of the JNK signal transduction pathway activated by proinflammatory cytokines. Genes. Dev..

[45] Sun Y., Zhang D., Guo X., Li W., Li C., Luo J., Zhou M., Xue L. (2019). MKK3 modulates JNK-dependent cell migration and invasion. Cell Death Dis..

[46] Huang C.Y., Tan T.H. (2012). DUSPs, to MAP kinases and beyond. Cell Biosci..

[47] Jeffrey K.L., Brummer T., Rolph M.S., Liu S.M., Callejas N.A., Grumont R.J., Gillieron C., Mackay F., Grey S., Camps M. (2006). Positive regulation of immune cell function and inflammatory responses by phosphatase PAC-1. Nat. Immunol..

[48] Kristiansen M., Hughes R., Patel P., Jacques T.S., Clark A.R., Ham J. (2010). Mkp1 is a c-Jun target gene that antagonizes JNK-dependent apoptosis in sympathetic neurons. J. Neurosci..

[49] Todd J.L., Rigas J.D., Rafty L.A., Denu J.M. (2002). Dual-specificity protein tyrosine phosphatase VHR down-regulates c-Jun N-terminal kinase (JNK). Oncogene.

[50] Ndong C., Landry R.P., Saha M., Romero-Sandoval E.A. (2014). Mitogen-activated protein kinase (MAPK) phosphatase-3 (MKP-3) displays a p-JNK-MAPK substrate preference in astrocytes in vitro. Neurosci. Lett..

[51] Muda M., Theodosiou A., Rodrigues N., Boschert U., Camps M., Gillieron C., Davies K., Ashworth A., Arkinstall S. (1996). The dual specificity phosphatases M3/6 and MKP-3 are highly selective for inactivation of distinct mitogen-activated protein kinases. J. Biol. Chem..

[52] Tanoue T., Yamamoto T., Maeda R., Nishida E. (2001). A novel MAPK phosphatase MKP-7 acts preferentially on JNK/SAPK and p38α and β MAPKs. J. Biol. Chem..

[53] Ye P., Xiang M., Liao H., Liu J., Luo H., Wang Y., Huang L., Chen M., Xia J. (2019). Dual-Specificity Phosphatase 9 Protects Against Nonalcoholic Fatty Liver Disease in Mice Through ASK1 Suppression. Hepatology.

[54] Yang C.Y., Li J.P., Chiu L.L., Lan J.L., Chen D.Y., Chuang H.C., Huang C.Y., Tan T.H. (2014). Dual-specificity phosphatase 14 (DUSP14/MKP6) negatively regulates TCR signaling by inhibiting TAB1 activation. J. Immunol..

[55] Zama T., Aoki R., Kamimoto T., Inoue K., Ikeda Y., Hagiwara M. (2002). A Novel Dual Specificity Phosphatase SKRP1 Interacts with the MAPK Kinase MKK7 and Inactivates the JNK MAPK Pathway: IMPLICATION FOR THE PRECISE REGULATION OF THE PARTICULAR MAPK PATHWAY*. J. Biol. Chem..

[56] Zama T., Aoki R., Kamimoto T., Inoue K., Ikeda Y., Hagiwara M. (2002). Scaffold role of a mitogen-activated protein kinase phosphatase, SKRP1, for the JNK signaling pathway. J. Biol. Chem..

[57] Takagaki K., Satoh T., Tanuma N., Masuda K., Takekawa M., Shima H., Kikuchi K. (2004). Characterization of a novel low-molecular-mass dual-specificity phosphatase-3 (LDP-3) that enhances activation of JNK and p38. Biochem. J..

[58] Elsby R., Kitteringham N.R., Goldring C.E., Lovatt C.A., Chamberlain M., Henderson C.J., Wolf C.R., Park B.K. (2003). Increased constitutive c-Jun N-terminal kinase signaling in mice lacking glutathione S-transferase Pi. J. Biol. Chem..

[59] Lee A.F., Ho D.K., Zanassi P., Walsh G.S., Kaplan D.R., Miller F.D. (2004). Evidence that DeltaNp73 promotes neuronal survival by p53-dependent and p53-independent mechanisms. J. Neurosci..

[60] Matsuyama M., Aizawa S., Shimono A. (2009). Sfrp controls apicobasal polarity and oriented cell division in developing gut epithelium. PLoS Genet..

[61] Sheridan D.L., Kong Y., Parker S.A., Dalby K.N., Turk B.E. (2008). Substrate Discrimination among Mitogen-activated Protein Kinases through Distinct Docking Sequence Motifs*. J. Biol. Chem..

[62] Björkblom B., Ostman N., Hongisto V., Komarovski V., Filén J.J., Nyman T.A., Kallunki T., Courtney M.J., Coffey E.T. (2005). Constitutively active cytoplasmic c-Jun N-terminal kinase 1 is a dominant regulator of dendritic architecture: Role of microtubule-associated protein 2 as an effector. J. Neurosci..

[63] Wang X., Song Q. (2018). Mst1 regulates post-infarction cardiac injury through the JNK-Drp1-mitochondrial fission pathway. Cell Mol. Biol. Lett..

[64] He Y.-L., Li J., Gong S.-H., Cheng X., Zhao M., Cao Y., Zhao T., Zhao Y.-Q., Fan M., Wu H.-T. (2022). BNIP3 phosphorylation by JNK1/2 promotes mitophagy via enhancing its stability under hypoxia. Cell Death Dis..

[65] Lei K., Davis R.J. (2003). JNK phosphorylation of Bim-related members of the Bcl2 family induces Bax-dependent apoptosis. Proc. Natl. Acad. Sci. USA.

[66] Hibi M., Lin A., Smeal T., Minden A., Karin M. (1993). Identification of an oncoprotein- and UV-responsive protein kinase that binds and potentiates the c-Jun activation domain. Genes. Dev..

[67] Buschmann T., Potapova O., Bar-Shira A., Ivanov V.N., Fuchs S.Y., Henderson S., Fried V.A., Minamoto T., Alarcon-Vargas D., Pincus M.R. (2001). Jun NH2-terminal kinase phosphorylation of p53 on Thr-81 is important for p53 stabilization and transcriptional activities in response to stress. Mol. Cell Biol..

[68] Noguchi K., Kitanaka C., Yamana H., Kokubu A., Mochizuki T., Kuchino Y. (1999). Regulation of c-Myc through Phosphorylation at Ser-62 and Ser-71 by c-Jun N-Terminal Kinase*. J. Biol. Chem..

[69] Zeke A., Misheva M., Reményi A., Bogoyevitch M.A. (2016). JNK Signaling: Regulation and Functions Based on Complex Protein-Protein Partnerships. Microbiol. Mol. Biol. Rev..

[70] Prakasam A., Ghose S., Oleinik N.V., Bethard J.R., Peterson Y.K., Krupenko N.I., Krupenko S.A. (2014). JNK1/2 regulate Bid by direct phosphorylation at Thr59 in response to ALDH1L1. Cell Death Dis..

[71] Tsuruta F., Sunayama J., Mori Y., Hattori S., Shimizu S., Tsujimoto Y., Yoshioka K., Masuyama N., Gotoh Y. (2004). JNK promotes Bax translocation to mitochondria through phosphorylation of 14-3-3 proteins. Embo J..

[72] Inoshita S., Takeda K., Hatai T., Terada Y., Sano M., Hata J., Umezawa A., Ichijo H. (2002). Phosphorylation and inactivation of myeloid cell leukemia 1 by JNK in response to oxidative stress. J. Biol. Chem..

[73] Zhang Y., Liu G., Dong Z. (2001). MSK1 and JNKs Mediate Phosphorylation of STAT3 in UVA-irradiated Mouse Epidermal JB6 Cells. J. Biol. Chem..

[74] Essers M.A.G., Weijzen S., de Vries-Smits A.M.M., Saarloos I., de Ruiter N.D., Bos J.L., Burgering B.M.T. (2004). FOXO transcription factor activation by oxidative stress mediated by the small GTPase Ral and JNK. EMBO J..

[75] Xia Z., Dickens M., Raingeaud J., Davis R.J., Greenberg M.E. (1995). Opposing effects of ERK and JNK-p38 MAP kinases on apoptosis. Science.

[76] Tournier C., Hess P., Yang D.D., Xu J., Turner T.K., Nimnual A., Bar-Sagi D., Jones S.N., Flavell R.A., Davis R.J. (2000). Requirement of JNK for stress-induced activation of the cytochrome c-mediated death pathway. Science.

[77] Behrens A., Sibilia M., Wagner E.F. (1999). Amino-terminal phosphorylation of c-Jun regulates stress-induced apoptosis and cellular proliferation. Nat. Genet..

[78] Zhang S., Lin Y., Kim Y.S., Hande M.P., Liu Z.G., Shen H.M. (2007). c-Jun N-terminal kinase mediates hydrogen peroxide-induced cell death via sustained poly(ADP-ribose) polymerase-1 activation. Cell Death Differ..

[79] Kuan C.Y., Yang D.D., Samanta Roy D.R., Davis R.J., Rakic P., Flavell R.A. (1999). The Jnk1 and Jnk2 protein kinases are required for regional specific apoptosis during early brain development. Neuron.

[80] Das M., Jiang F., Sluss H.K., Zhang C., Shokat K.M., Flavell R.A., Davis R.J. (2007). Suppression of p53-dependent senescence by the JNK signal transduction pathway. Proc. Natl. Acad. Sci. USA.

[81] Wisdom R., Johnson R.S., Moore C. (1999). c-Jun regulates cell cycle progression and apoptosis by distinct mechanisms. Embo J..

[82] Johnson R., Spiegelman B., Hanahan D., Wisdom R. (1996). Cellular transformation and malignancy induced by ras require c-jun. Mol. Cell Biol..

[83] Ventura J.-J., Hübner A., Zhang C., Flavell R.A., Shokat K.M., Davis R.J. (2006). Chemical Genetic Analysis of the Time Course of Signal Transduction by JNK. Mol. Cell.

[84] Brown M., Strudwick N., Suwara M., Sutcliffe L.K., Mihai A.D., Ali A.A., Watson J.N., Schröder M. (2016). An initial phase of JNK activation inhibits cell death early in the endoplasmic reticulum stress response. J. Cell Sci..

[85] Jung T.W., Lee M.W., Lee Y.J., Kim S.M. (2012). Metformin prevents endoplasmic reticulum stress-induced apoptosis through AMPK-PI3K-c-Jun NH2 pathway. Biochem. Biophys. Res. Commun..

[86] Marshall C. (1995). Specificity of receptor tyrosine kinase signaling: Transient versus sustained extracellular signal-regulated kinase activation. Cell.

[87] Jones S.M., Kazlauskas A. (2001). Growth-factor-dependent mitogenesis requires two distinct phases of signalling. Nat. Cell Biol..

[88] Katsura Y., Kubota H., Kunida K., Kanno A., Kuroda S., Ozawa T. (2015). An optogenetic system for interrogating the temporal dynamics of Akt. Sci. Rep..

[89] Regot S., Hughey J.J., Bajar B.T., Carrasco S., Covert M.W. (2014). High-sensitivity measurements of multiple kinase activities in live single cells. Cell.

[90] Taylor R.J., Falconnet D., Niemistö A., Ramsey S.A., Prinz S., Shmulevich I., Galitski T., Hansen C.L. (2009). Dynamic analysis of MAPK signaling using a high-throughput microfluidic single-cell imaging platform. Proc. Natl. Acad. Sci. USA.

[91] Jedariforoughi A., Burke R., Chesak A., Gonzalez Hernandez J.L., Hanson R.L. (2025). JNK activation dynamics drive distinct gene expression patterns over time mediated by mRNA stability. npj Syst. Biol. Appl..

[92] Harvey C.D., Ehrhardt A.G., Cellurale C., Zhong H., Yasuda R., Davis R.J., Svoboda K. (2008). A genetically encoded fluorescent sensor of ERK activity. Proc. Natl. Acad. Sci. USA.

[93] Fosbrink M., Aye-Han N.-N., Cheong R., Levchenko A., Zhang J. (2010). Visualization of JNK activity dynamics with a genetically encoded fluorescent biosensor. Proc. Natl. Acad. Sci. USA.

[94] Didan Y., Ghomlaghi M., Nguyen L.K., Ng D.C.H. (2024). Stress pathway outputs are encoded by pH-dependent clustering of kinase components. Nat. Commun..

[95] Miura H., Kondo Y., Matsuda M., Aoki K. (2018). Cell-to-Cell Heterogeneity in p38-Mediated Cross-Inhibition of JNK Causes Stochastic Cell Death. Cell Rep..

[96] Hanson R.L., Batchelor E. (2022). Coordination of MAPK and p53 dynamics in the cellular responses to DNA damage and oxidative stress. Mol. Syst. Biol..

[97] Netterfield T.S., Ostheimer G.J., Tentner A.R., Joughin B.A., Dakoyannis A.M., Sharma C.D., Sorger P.K., Janes K.A., Lauffenburger D.A., Yaffe M.B. (2023). Biphasic JNK-Erk signaling separates the induction and maintenance of cell senescence after DNA damage induced by topoisomerase II inhibition. Cell Syst..

[98] Hisashi M., Takanori N., Yuji K., Ryosuke H., Youngmin C., Toru K., Hiroyuki T., Takashi S., Mutsuhiro T. (2026). Spatiotemporal regulation of MKK4 dictates switch-like JNK activation and binary cell-fate decisions. Nat. Commun..

[99] Leavesley S.J., Rich T.C. (2016). Overcoming limitations of FRET measurements. Cytom. A.

[100] Hoffman T.E., Tian C., Nangia V., Yang C., Regot S., Gerosa L., Spencer S.L. (2025). CDK2 activity crosstalk on the ERK kinase translocation reporter can be resolved computationally. Cell Syst..

[101] Tsai S.-J., Gong Y., Dabbs A., Zahra F., Xu J., Geske A., Caterina M.J., Gould S.J. (2025). Enhanced kinase translocation reporters for simultaneous real-time measurement of PKA, ERK, and calcium. J. Biol. Chem..

[102] Jimenez A., Lu D., Kalocsay M., Berberich M.J., Balbi P., Jambhekar A., Lahav G. (2022). Time-series transcriptomics and proteomics reveal alternative modes to decode p53 oscillations. Mol. Syst. Biol..

[103] Yang Z., Hanson R.L., Batchelor E. (2021). Progress and challenges in understanding the regulation and function of p53 dynamics. Biochem. Soc. Trans..

[104] Hanson R.L., Porter J.R., Batchelor E. (2019). Protein stability of p53 targets determines their temporal expression dynamics in response to p53 pulsing. J. Cell Biol..

[105] Porter J.R., Fisher B.E., Batchelor E. (2016). p53 Pulses Diversify Target Gene Expression Dynamics in an mRNA Half-Life-Dependent Manner and Delineate Co-regulated Target Gene Subnetworks. Cell Syst..

[106] Hafner A., Stewart-Ornstein J., Purvis J.E., Forrester W.C., Bulyk M.L., Lahav G. (2017). p53 pulses lead to distinct patterns of gene expression albeit similar DNA-binding dynamics. Nat. Struct. Mol. Biol..

[107] Waudby C.A., Alvarez-Teijeiro S., Josue Ruiz E., Suppinger S., Pinotsis N., Brown P.R., Behrens A., Christodoulou J., Mylona A. (2022). An intrinsic temporal order of c-JUN N-terminal phosphorylation regulates its activity by orchestrating co-factor recruitment. Nat. Commun..

[108] Harton M.D., Koh W.S., Bunker A.D., Singh A., Batchelor E. (2019). p53 pulse modulation differentially regulates target gene promoters to regulate cell fate decisions. Mol. Syst. Biol..

[109] Hansen A.S., O’Shea E.K. (2013). Promoter decoding of transcription factor dynamics involves a trade-off between noise and control of gene expression. Mol. Syst. Biol..

[110] Ha J., Kang E., Seo J., Cho S. (2019). Phosphorylation Dynamics of JNK Signaling: Effects of Dual-Specificity Phosphatases (DUSPs) on the JNK Pathway. Int. J. Mol. Sci..

[111] Win S., Than T., Kaplowitz N. (2018). The Regulation of JNK Signaling Pathways in Cell Death through the Interplay with Mitochondrial SAB and Upstream Post-Translational Effects. Int. J. Mol. Sci..

[112] Engström W., Ward A., Moorwood K. (2010). The role of scaffold proteins in JNK signalling. Cell Prolif..

[113] Yasuda J., Whitmarsh A.J., Cavanagh J., Sharma M., Davis R.J. (1999). The JIP group of mitogen-activated protein kinase scaffold proteins. Mol. Cell Biol..

[114] Dajas-Bailador F., Jones E.V., Whitmarsh A.J. (2008). The JIP1 scaffold protein regulates axonal development in cortical neurons. Curr. Biol..

[115] Meyer D., Liu A., Margolis B. (1999). Interaction of c-Jun Amino-terminal Kinase Interacting Protein-1 with p190 rhoGEF and Its Localization in Differentiated Neurons*. J. Biol. Chem..

[116] Morel C., Standen C.L., Jung D.Y., Gray S., Ong H., Flavell R.A., Kim J.K., Davis R.J. (2010). Requirement of JIP1-mediated c-Jun N-terminal kinase activation for obesity-induced insulin resistance. Mol. Cell Biol..

[117] Roessler R., Goldmann J., Shivalila C., Jaenisch R. (2018). JIP2 haploinsufficiency contributes to neurodevelopmental abnormalities in human pluripotent stem cell-derived neural progenitors and cortical neurons. Life Sci. Alliance.

[118] Watt D., Dixit R., Cavalli V. (2015). JIP3 Activates Kinesin-1 Motility to Promote Axon Elongation. J. Biol. Chem..

[119] Caswell P.T., Dickens M. (2018). JIP3 localises to exocytic vesicles and focal adhesions in the growth cones of differentiated PC12 cells. Mol. Cell Biochem..

[120] Kelkar N., Standen C.L., Davis R.J. (2005). Role of the JIP4 scaffold protein in the regulation of mitogen-activated protein kinase signaling pathways. Mol. Cell Biol..

[121] Lee C.M., Onésime D., Reddy C.D., Dhanasekaran N., Reddy E.P. (2002). JLP: A scaffolding protein that tethers JNK/p38MAPK signaling modules and transcription factors. Proc. Natl. Acad. Sci. USA.

[122] Jagadish N., Rana R., Mishra D., Garg M., Chaurasiya D., Hasegawa A., Koyama K., Suri A. (2005). Immunogenicity and contraceptive potential of recombinant human sperm associated antigen (SPAG9). J. Reprod. Immunol..

[123] Harding T.C., Xue L., Bienemann A., Haywood D., Dickens M., Tolkovsky A.M., Uney J.B. (2001). Inhibition of JNK by Overexpression of the JNK Binding Domain of JIP-1 Prevents Apoptosis in Sympathetic Neurons*. J. Biol. Chem..

[124] Wasserman T., Katsenelson K., Daniliuc S., Hasin T., Choder M., Aronheim A. (2010). A novel c-Jun N-terminal kinase (JNK)-binding protein WDR62 is recruited to stress granules and mediates a nonclassical JNK activation. Mol. Biol. Cell.

[125] Mahmood S., Ahmad W., Hassan M.J. (2011). Autosomal recessive primary microcephaly (MCPH): Clinical manifestations, genetic heterogeneity and mutation continuum. Orphanet J. Rare Dis..

[126] Zhi Y., Zhou X., Yu J., Yuan L., Zhang H., Ng D.C.H., Xu Z., Xu D. (2021). Pathophysiological Significance of WDR62 and JNK Signaling in Human Diseases. Front. Cell Dev. Biol..

[127] Bogoyevitch M.A., Yeap Y.Y.C., Qu Z., Ngoei K.R., Yip Y.Y., Zhao T.T., Heng J.I., Ng D.C.H. (2012). WD40-repeat protein 62 is a JNK-phosphorylated spindle pole protein required for spindle maintenance and timely mitotic progression. J. Cell Sci..

[128] Xu D., Zhang F., Wang Y., Sun Y., Xu Z. (2014). Microcephaly-associated protein WDR62 regulates neurogenesis through JNK1 in the developing neocortex. Cell Rep..

[129] Gutierrez G.J., Tsuji T., Cross J.V., Davis R.J., Templeton D.J., Jiang W., Ronai Z.e.A. (2010). JNK-mediated Phosphorylation of Cdc25C Regulates Cell Cycle Entry and G2/M DNA Damage Checkpoint*. J. Biol. Chem..

[130] Gutierrez G.J., Tsuji T., Chen M., Jiang W., Ronai Z.e.A. (2010). Interplay between Cdh1 and JNK activity during the cell cycle. Nat. Cell Biol..

[131] Sahana T.G., Chase K.J., Liu F., Lloyd T.E., Rossoll W., Zhang K. (2023). c-Jun N-Terminal Kinase Promotes Stress Granule Assembly and Neurodegeneration in C9orf72-Mediated ALS and FTD. J. Neurosci..

[132] Xu Z., Kukekov N.V., Greene L.A. (2003). POSH acts as a scaffold for a multiprotein complex that mediates JNK activation in apoptosis. Embo J..

[133] Karkkainen S., van der Linden M., Renkema G.H. (2010). POSH2 is a RING finger E3 ligase with Rac1 binding activity through a partial CRIB domain. FEBS Lett..

[134] Kukekov N.V., Xu Z., Greene L.A. (2006). Direct Interaction of the Molecular Scaffolds POSH and JIP Is Required for Apoptotic Activation of JNKs*. J. Biol. Chem..

[135] Yang T., Sun Y., Zhang F., Zhu Y., Shi L., Li H., Xu Z. (2012). POSH Localizes Activated Rac1 to Control the Formation of Cytoplasmic Dilation of the Leading Process and Neuronal Migration. Cell Rep..

[136] Kim G.-H., Park E., Kong Y.-Y., Han J.-K. (2006). Novel function of POSH, a JNK scaffold, as an E3 ubiquitin ligase for the Hrs stability on early endosomes. Cell. Signal..

[137] Wilhelm M., Kukekov N.V., Schmit T.L., Biagas K.V., Sproul A.A., Gire S., Maes M.E., Xu Z., Greene L.A. (2012). Sh3rf2/POSHER protein promotes cell survival by ring-mediated proteasomal degradation of the c-Jun N-terminal kinase scaffold POSH (Plenty of SH3s) protein. J. Biol. Chem..

[138] Wilhelm M., Kukekov N.V., Xu Z., Greene L.A. (2007). Identification of POSH2, a novel homologue of the c-Jun N-terminal kinase scaffold protein POSH. Dev. Neurosci..

[139] Jean-Charles P.Y., Kaur S., Shenoy S.K. (2017). G Protein-Coupled Receptor Signaling Through β-Arrestin-Dependent Mechanisms. J. Cardiovasc. Pharmacol..

[140] McDonald P.H., Chow C.-W., Miller W.E., Laporte S.A., Field M.E., Lin F.-T., Davis R.J., Lefkowitz R.J. (2000). β-Arrestin 2: A Receptor-Regulated MAPK Scaffold for the Activation of JNK3. Science.

[141] Guo C., Whitmarsh A.J. (2008). The beta-arrestin-2 scaffold protein promotes c-Jun N-terminal kinase-3 activation by binding to its nonconserved N terminus. J. Biol. Chem..

[142] Scott M.G., Le Rouzic E., Perianin A., Pierotti V., Enslen H., Benichou S., Marullo S., Benmerah A. (2002). Differential nucleocytoplasmic shuttling of beta-arrestins. Characterization of a leucine-rich nuclear export signal in beta-arrestin2. J. Biol. Chem..

[143] Ahmed M.R., Zheng C., Dunning J.L., Ahmed M.S., Ge C., Pair F.S., Gurevich V.V., Gurevich E.V. (2024). Arrestin-3-assisted activation of JNK3 mediates dopaminergic behavioral sensitization. Cell Rep. Med..

[144] Zhu L., Almaça J., Dadi P.K., Hong H., Sakamoto W., Rossi M., Lee R.J., Vierra N.C., Lu H., Cui Y. (2017). β-arrestin-2 is an essential regulator of pancreatic β-cell function under physiological and pathophysiological conditions. Nat. Commun..

[145] Slee J.B., Lowe-Krentz L.J. (2013). Actin realignment and cofilin regulation are essential for barrier integrity during shear stress. J. Cell Biochem..

[146] Gordon E.A., Whisenant T.C., Zeller M., Kaake R.M., Gordon W.M., Krotee P., Patel V., Huang L., Baldi P., Bardwell L. (2013). Combining docking site and phosphosite predictions to find new substrates: Identification of smoothelin-like-2 (SMTNL2) as a c-Jun N-terminal kinase (JNK) substrate. Cell Signal.

[147] Bjorkblom B., Padzik A., Mohammad H., Westerlund N., Komulainen E., Hollos P., Parviainen L., Papageorgiou A.C., Iljin K., Kallioniemi O. (2012). c-Jun N-terminal kinase phosphorylation of MARCKSL1 determines actin stability and migration in neurons and in cancer cells. Mol. Cell Biol..

[148] Lee M.H., Koria P., Qu J., Andreadis S.T. (2009). JNK phosphorylates beta-catenin and regulates adherens junctions. FASEB J..

[149] Eyers C.E., McNeill H., Knebel A., Morrice N., Arthur S.J., Cuenda A., Cohen P. (2005). The phosphorylation of CapZ-interacting protein (CapZIP) by stress-activated protein kinases triggers its dissociation from CapZ. Biochem. J..

[150] Huang C., Rajfur Z., Borchers C., Schaller M.D., Jacobson K. (2003). JNK phosphorylates paxillin and regulates cell migration. Nature.

[151] Chatzifrangkeskou M., Kouis P., Skourides P.A. (2023). JNK regulates ciliogenesis through the interflagellar transport complex and actin networks. J. Cell Biol..

[152] Satake T., Otsuki K., Banba Y., Suenaga J., Hirano H., Yamanaka Y., Ohno S., Hirai S. (2013). The interaction of Kinesin-1 with its adaptor protein JIP1 can be regulated via proteins binding to the JIP1-PTB domain. BMC Cell Biol..

[153] Daire V., Giustiniani J., Leroy-Gori I., Quesnoit M., Drevensek S., Dimitrov A., Perez F., Pous C. (2009). Kinesin-1 regulates microtubule dynamics via a c-Jun N-terminal kinase-dependent mechanism. J. Biol. Chem..

[154] Benoit B., Baillet A., Poüs C. (2021). Cytoskeleton and Associated Proteins: Pleiotropic JNK Substrates and Regulators. Int. J. Mol. Sci..

[155] Chang L., Jones Y., Ellisman M.H., Goldstein L.S., Karin M. (2003). JNK1 is required for maintenance of neuronal microtubules and controls phosphorylation of microtubule-associated proteins. Dev. Cell.

[156] Yoshida H., Hastie C.J., McLauchlan H., Cohen P., Goedert M. (2004). Phosphorylation of microtubule-associated protein tau by isoforms of c-Jun N-terminal kinase (JNK). J. Neurochem..

[157] Padzik A., Deshpande P., Hollos P., Franker M., Rannikko E.H., Cai D., Prus P., Mågård M., Westerlund N., Verhey K.J. (2016). KIF5C S176 Phosphorylation Regulates Microtubule Binding and Transport Efficiency in Mammalian Neurons. Front. Cell Neurosci..

[158] Bharat V., Siebrecht M., Burk K., Ahmed S., Reissner C., Kohansal-Nodehi M., Steubler V., Zweckstetter M., Ting J.T., Dean C. (2017). Capture of Dense Core Vesicles at Synapses by JNK-Dependent Phosphorylation of Synaptotagmin-4. Cell Rep..

[159] Javed E., Thangavel C., Frara N., Singh J., Mohanty I., Hypolite J., Birbe R., Braverman A.S., Den R.B., Rattan S. (2020). Increased expression of desmin and vimentin reduces bladder smooth muscle contractility via JNK2. Faseb J..

[160] He T., Stepulak A., Holmström T.H., Omary M.B., Eriksson J.E. (2002). The intermediate filament protein keratin 8 is a novel cytoplasmic substrate for c-Jun N-terminal kinase. J. Biol. Chem..

[161] Park M.K., Lee H.J., Shin J., Noh M., Kim S.Y., Lee C.H. (2011). Novel participation of transglutaminase-2 through c-Jun N-terminal kinase activation in sphingosylphosphorylcholine-induced keratin reorganization of PANC-1 cells. Biochim. Biophys. Acta.

[162] Giasson B.I., Mushynski W.E. (1996). Aberrant Stress-induced Phosphorylation of Perikaryal Neurofilaments*. J. Biol. Chem..

[163] Boubriak I.I., Malhas A.N., Drozdz M.M., Pytowski L., Vaux D.J. (2017). Stress-induced release of Oct-1 from the nuclear envelope is mediated by JNK phosphorylation of lamin B1. PLoS ONE.

[164] Mustafa M., Ahmad R., Tantry I.Q., Ahmad W., Siddiqui S., Alam M., Abbas K., Moinuddin, Hassan M.I., Habib S. (2024). Apoptosis: A Comprehensive Overview of Signaling Pathways, Morphological Changes, and Physiological Significance and Therapeutic Implications. Cells.

[165] Wiltshire C., Matsushita M., Tsukada S., Gillespie D.A., May G.H. (2002). A new c-Jun N-terminal kinase (JNK)-interacting protein, Sab (SH3BP5), associates with mitochondria. Biochem. J..

[166] Soberanes S., Urich D., Baker C.M., Burgess Z., Chiarella S.E., Bell E.L., Ghio A.J., De Vizcaya-Ruiz A., Liu J., Ridge K.M. (2009). Mitochondrial complex III-generated oxidants activate ASK1 and JNK to induce alveolar epithelial cell death following exposure to particulate matter air pollution. J. Biol. Chem..

[167] Wei M.C., Lindsten T., Mootha V.K., Weiler S., Gross A., Ashiya M., Thompson C.B., Korsmeyer S.J. (2000). tBID, a membrane-targeted death ligand, oligomerizes BAK to release cytochrome c. Genes. Dev..

[168] Chambers J.W., Howard S., LoGrasso P.V. (2013). Blocking c-Jun N-terminal kinase (JNK) translocation to the mitochondria prevents 6-hydroxydopamine-induced toxicity in vitro and in vivo. J. Biol. Chem..

[169] Chambers J.W., Cherry L., Laughlin J.D., Figuera-Losada M., Lograsso P.V. (2011). Selective inhibition of mitochondrial JNK signaling achieved using peptide mimicry of the Sab kinase interacting motif-1 (KIM1). ACS Chem. Biol..

[170] Urano F., Wang X., Bertolotti A., Zhang Y., Chung P., Harding H.P., Ron D. (2000). Coupling of stress in the ER to activation of JNK protein kinases by transmembrane protein kinase IRE1. Science.

[171] Verma G., Bhatia H., Datta M. (2013). JNK1/2 regulates ER-mitochondrial Ca^2+^ cross-talk during IL-1β-mediated cell death in RINm5F and human primary β-cells. Mol. Biol. Cell.

[172] Larrañaga-SanMiguel A., Bengoa-Vergniory N., Flores-Romero H. (2025). Crosstalk between mitochondria–ER contact sites and the apoptotic machinery as a novel health meter. Trends Cell Biol..

[173] Nordgaard C., Tollenaere M.A.X., Val A.M.D., Bekker-Jensen D.B., Blasius M., Olsen J.V., Bekker-Jensen S. (2021). Regulation of the Golgi Apparatus by p38 and JNK Kinases during Cellular Stress Responses. Int. J. Mol. Sci..

[174] Harada T., Matsuzaki O., Hayashi H., Sugano S., Matsuda A., Nishida E. (2003). AKRL1 and AKRL2 activate the JNK pathway. Genes. Cells.

[175] Singaraja R.R., Hadano S., Metzler M., Givan S., Wellington C.L., Warby S., Yanai A., Gutekunst C.A., Leavitt B.R., Yi H. (2002). HIP14, a novel ankyrin domain-containing protein, links huntingtin to intracellular trafficking and endocytosis. Hum. Mol. Genet..

[176] Biggi S., Buccarello L., Sclip A., Lippiello P., Tonna N., Rumio C., Di Marino D., Miniaci M.C., Borsello T. (2017). Evidence of Presynaptic Localization and Function of the c-Jun N-Terminal Kinase. Neural Plast..

[177] Sclip A., Tozzi A., Abaza A., Cardinetti D., Colombo I., Calabresi P., Salmona M., Welker E., Borsello T. (2014). c-Jun N-terminal kinase has a key role in Alzheimer disease synaptic dysfunction in vivo. Cell Death Dis..

[178] Decker C.J., Parker R. (2012). P-bodies and stress granules: Possible roles in the control of translation and mRNA degradation. Cold Spring Harb. Perspect. Biol..

[179] Cargnello M., Tcherkezian J., Dorn J.F., Huttlin E.L., Maddox P.S., Gygi S.P., Roux P.P. (2012). Phosphorylation of the eukaryotic translation initiation factor 4E-transporter (4E-T) by c-Jun N-terminal kinase promotes stress-dependent P-body assembly. Mol. Cell Biol..

[180] Rzeczkowski K., Beuerlein K., Müller H., Dittrich-Breiholz O., Schneider H., Kettner-Buhrow D., Holtmann H., Kracht M. (2011). c-Jun N-terminal kinase phosphorylates DCP1a to control formation of P bodies. J. Cell Biol..

[181] Maik-Rachline G., Zehorai E., Hanoch T., Blenis J., Seger R. (2018). The nuclear translocation of the kinases p38 and JNK promotes inflammation-induced cancer. Sci. Signal..

[182] Hu M.C., Qiu W.R., Wang Y.P. (1997). JNK1, JNK2 and JNK3 are p53 N-terminal serine 34 kinases. Oncogene.

[183] Zhang T., Inesta-Vaquera F., Niepel M., Zhang J., Ficarro S.B., Machleidt T., Xie T., Marto J.A., Kim N., Sim T. (2012). Discovery of potent and selective covalent inhibitors of JNK. Chem. Biol..

[184] van der Velden J.L.J., Ye Y., Nolin J.D., Hoffman S.M., Chapman D.G., Lahue K.G., Abdalla S., Chen P., Liu Y., Bennett B. (2016). JNK inhibition reduces lung remodeling and pulmonary fibrotic systemic markers. Clin. Transl. Med..

[185] Staecker H., Jokovic G., Karpishchenko S., Kienle-Gogolok A., Krzyzaniak A., Lin C.D., Navratil P., Tzvetkov V., Wright N., Meyer T. (2019). Efficacy and Safety of AM-111 in the Treatment of Acute Unilateral Sudden Deafness-A Double-blind, Randomized, Placebo-controlled Phase 3 Study. Otol. Neurotol..

[186] Mattos W., Khalil N., Spencer L.G., Bonella F., Folz R.J., Rolf J.D., Mogulkoc N., Lancaster L.H., Jenkins R.G., Lynch D.A. (2024). Phase 2, Double-Blind, Placebo-controlled Trial of a c-Jun N-Terminal Kinase Inhibitor in Idiopathic Pulmonary Fibrosis. Am. J. Respir. Crit. Care Med..

[187] Chambers J.W., Pachori A., Howard S., Iqbal S., LoGrasso P.V. (2013). Inhibition of JNK mitochondrial localization and signaling is protective against ischemia/reperfusion injury in rats. J. Biol. Chem..

[188] Aikawa Y., Morimoto K., Yamamoto T., Chaki H., Hashiramoto A., Narita H., Hirono S., Shiozawa S. (2008). Treatment of arthritis with a selective inhibitor of c-Fos/activator protein-1. Nat. Biotechnol..

[189] Adler V., Pincus M.R., Minamoto T., Fuchs S.Y., Bluth M.J., Brandt-Rauf P.W., Friedman F.K., Robinson R.C., Chen J.M., Wang X.W. (1997). Conformation-dependent phosphorylation of p53. Proc. Natl. Acad. Sci. USA.

[190] Oleinik N.V., Krupenko N.I., Krupenko S.A. (2007). Cooperation between JNK1 and JNK2 in activation of p53 apoptotic pathway. Oncogene.

[191] Patel J.A., Rageul J., Lo N., Nandi A., Zezelic C., Lee C.T., Khan A., Kim H. (2025). The DNA-PKcs/JNK/p53 pathway underlies changes in cell fate decision toward death during DNA replication catastrophe. Nucleic Acids Res..

[192] Fuchs S.Y., Adler V., Buschmann T., Yin Z., Wu X., Jones S.N., Ronai Z. (1998). JNK targets p53 ubiquitination and degradation in nonstressed cells. Genes. Dev..

[193] Schreiber M., Kolbus A., Piu F., Szabowski A., Möhle-Steinlein U., Tian J., Karin M., Angel P., Wagner E.F. (1999). Control of cell cycle progression by c-Jun is p53 dependent. Genes. Dev..

[194] Gowda P.S., Zhou F., Chadwell L.V., McEwen D.G. (2012). p53 binding prevents phosphatase-mediated inactivation of diphosphorylated c-Jun N-terminal kinase. J. Biol. Chem..

[195] Batchelor E., Loewer A., Mock C., Lahav G. (2011). Stimulus-dependent dynamics of p53 in single cells. Mol. Syst. Biol..

[196] Batchelor E., Mock C.S., Bhan I., Loewer A., Lahav G. (2008). Recurrent initiation: A mechanism for triggering p53 pulses in response to DNA damage. Mol. Cell.

[197] Lahav G., Rosenfeld N., Sigal A., Geva-Zatorsky N., Levine A.J., Elowitz M.B., Alon U. (2004). Dynamics of the p53-Mdm2 feedback loop in individual cells. Nat. Genet..

[198] Reyes J., Chen J.Y., Stewart-Ornstein J., Karhohs K.W., Mock C.S., Lahav G. (2018). Fluctuations in p53 Signaling Allow Escape from Cell-Cycle Arrest. Mol. Cell.

[199] Hafner A., Reyes J., Stewart-Ornstein J., Tsabar M., Jambhekar A., Lahav G. (2020). Quantifying the Central Dogma in the p53 Pathway in Live Single Cells. Cell Syst..

[200] Tang F., Tang G., Xiang J., Dai Q., Rosner M.R., Lin A. (2002). The absence of NF-kappaB-mediated inhibition of c-Jun N-terminal kinase activation contributes to tumor necrosis factor alpha-induced apoptosis. Mol. Cell Biol..

[201] Tang G., Minemoto Y., Dibling B., Purcell N.H., Li Z., Karin M., Lin A. (2001). Inhibition of JNK activation through NF-kappaB target genes. Nature.

[202] Zhang Q., Gupta S., Schipper D.L., Kowalczyk G.J., Mancini A.E., Faeder J.R., Lee R.E.C. (2017). NF-κB Dynamics Discriminate between TNF Doses in Single Cells. Cell Syst..

[203] Tay S., Hughey J.J., Lee T.K., Lipniacki T., Quake S.R., Covert M.W. (2010). Single-cell NF-kappaB dynamics reveal digital activation and analogue information processing. Nature.

[204] Cheng C.S., Behar M.S., Suryawanshi G.W., Feldman K.E., Spreafico R., Hoffmann A. (2017). Iterative Modeling Reveals Evidence of Sequential Transcriptional Control Mechanisms. Cell Syst..

[205] Rodríguez-Martínez J.A., Reinke A.W., Bhimsaria D., Keating A.E., Ansari A.Z. (2017). Combinatorial bZIP dimers display complex DNA-binding specificity landscapes. eLife.

[206] Bakiri L., Matsuo K., Wisniewska M., Wagner E.F., Yaniv M. (2002). Promoter specificity and biological activity of tethered AP-1 dimers. Mol. Cell Biol..

[207] Cook S.J., Aziz N., McMahon M. (1999). The repertoire of fos and jun proteins expressed during the G1 phase of the cell cycle is determined by the duration of mitogen-activated protein kinase activation. Mol. Cell Biol..

[208] Mehic D., Bakiri L., Ghannadan M., Wagner E.F., Tschachler E. (2005). Fos and Jun Proteins Are Specifically Expressed During Differentiation of Human Keratinocytes. J. Investig. Dermatol..

[209] Willsey H.R., Zheng X., Carlos Pastor-Pareja J., Willsey A.J., Beachy P.A., Xu T. (2016). Localized JNK signaling regulates organ size during development. eLife.

[210] Bosch M., Serras F., Martín-Blanco E., Baguñà J. (2005). JNK signaling pathway required for wound healing in regenerating Drosophila wing imaginal discs. Dev. Biol..

[211] Becker C.J., Cigliola V., Gillotay P., Rich A., De Simone A., Han Y., Di Talia S., Poss K.D. (2023). In toto imaging of glial JNK signaling during larval zebrafish spinal cord regeneration. Development.

[212] Guntas G., Hallett R.A., Zimmerman S.P., Williams T., Yumerefendi H., Bear J.E., Kuhlman B. (2015). Engineering an improved light-induced dimer (iLID) for controlling the localization and activity of signaling proteins. Proc. Natl. Acad. Sci. USA.

[213] Cho K.F., Branon T.C., Udeshi N.D., Myers S.A., Carr S.A., Ting A.Y. (2020). Proximity labeling in mammalian cells with TurboID and split-TurboID. Nat. Protoc..

[214] Dumrongprechachan V., Salisbury R.B., Soto G., Kumar M., MacDonald M.L., Kozorovitskiy Y. (2021). Cell-type and subcellular compartment-specific APEX2 proximity labeling reveals activity-dependent nuclear proteome dynamics in the striatum. Nat. Commun..

